# Hexagonal Au Nanostructure SERS Metasurface for AI-Driven Detection of Pesticide Residues in Real Food Samples

**DOI:** 10.1021/acsanm.6c00433

**Published:** 2026-04-10

**Authors:** Sümeyra Vural Kaymaz, Mustafa Özen, Süleyman Çelik, Selim Tanrıseven, Elmas Eva Öktem Olgun, Oltan Canlı, Barış Güzel, Yunus Sarıkaya, Hasan Kurt, Meral Yüce

**Affiliations:** † Department of Molecular Biology, Genetics, and Bioengineering, Faculty of Engineering and Natural Sciences, 52991 Sabanci University, 34956 Istanbul, Turkey; ‡ Department of Computer Science, Faculty of Engineering and Natural Sciences, Sabanci University, 34956 Istanbul, Turkey; § SUNUM Nanotechnology Research and Application Centre, Sabanci University, Istanbul 34956, Turkey; ∥ Climate Studies and Water Management Research Group, Climate and Life Sciences Vice Presidency, TUBITAK Marmara Research Center, 41470 Gebze, Kocaeli, Turkey; ⊥ Pusula AI, Oakland, California 94611, United States; # Department of Biomedical Engineering, School of Engineering and Natural Sciences, Istanbul Medipol University, Istanbul 34810, Turkey; ¶ Research Institute for Health Sciences and Technologies (SABITA), Istanbul Medipol University, Istanbul 34810, Turkey; ∇ Department of Bioengineering, 4615 Imperial College London, South Kensington Campus, London SW7 2AZ, U.K.

**Keywords:** SERS, honeycomb array, metal−insulator−metal metasurface, food matrix, machine learning, fungicide, insecticide, deep feed-forward

## Abstract

Pesticide residues in food remain a major threat to human health and ecosystems, yet routine monitoring still relies on centralized, multistep analytical workflows which are poorly suited to rapid and field-deployable detection. In this work, we introduce a rationally designed hexagonal honeycomb metal–insulator–metal (MIM) plasmonic metasurface which functions as a robust, wafer-scale surface/plasmon-enhanced Raman spectroscopy (SERS) platform for pesticide quantification in real food matrices. The MIM honeycomb architecture simultaneously creates highly concentrated electromagnetic hotspots at the excitation wavelength and a plasmonic antenna effect that radiates the Stokes-shifted Raman signals back, effectively multiplying the Raman signal and enabling sensitive detection of multiple fungicides and insecticides directly in cucumber extracts. We show that characteristic vibrational fingerprints can be reliably captured for several representative pesticides (metalaxyl, boscalid, famoxadone, thiamethoxam, etoxazole, cypermethrin) across realistic concentration ranges and in the presence of complex matrix backgrounds, achieving subppm limits of detection that approach or fall below current regulatory maximum residue limits. To convert raw spectra into actionable readouts, we integrate our process flow with a deep feed-forward (DFF) artificial intelligence model pipeline that performs automated spectral preprocessing and supervised learning for both pesticide identification and residue-level classification with respect to regulatory thresholds. This AI-enabled MIM-SERS platform establishes a generalizable route toward compact, high-throughput instruments for multiresidue pesticide surveillance in real food samples, with broader implications for molecular diagnostics and environmental monitoring.

## Graphical abstract

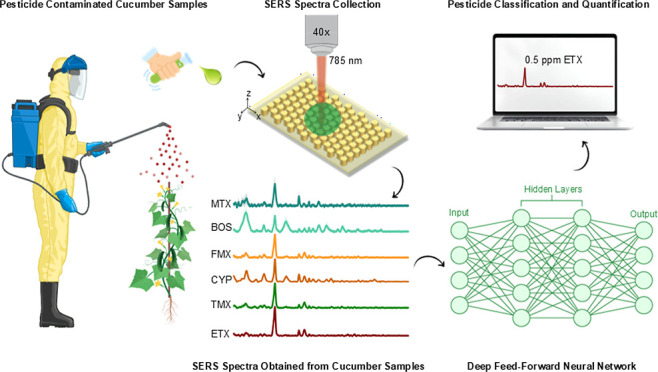

## 1 Introduction

Pesticides are chemical substances widely used to increase agricultural productivity and control infestations of pests; however, their widespread and often uncontrolled application has raised significant concerns regarding food safety and environmental health. A variety of pesticides are utilized in industry, including insecticides, rodenticides, herbicides, fungicides, biocides, and others. These compounds are classified according to their chemical structure and the types of pests they target. The two most used synthetic pesticide classes in agriculture are insecticides and fungicides.^1^ Although these compounds are effective in reducing agricultural losses, they can accumulate as residues on plants and pose long-term health risks to humans. Chronic and acute exposure to pesticide residues has been associated with a range of adverse health effects, including neurological disorders, endocrine system damage, cancer, as well as acute poisoning and chronic disorders. The World Health Organization (WHO) reports that pesticides cause approximately 3 million cases of poisoning and 220,000 fatalities annually. Moreover, pesticides affect not only humans but also the environment, disrupting the balance in ecosystems and causing a decline in biodiversity. Therefore, the development of sensors that can quickly and accurately detect pesticide residues in food is of immense importance for ensuring food safety and a healthier future.^2^

Early detection not only facilitates compliance with legal regulations but also protects the overall health of human populations and agricultural ecosystems by enabling timely preventive measures. Due to the potential adverse effects and unpredictable consequences of these compounds on the environment, careful application and continuous monitoring are crucial.^3^ Strict adherence to safety protocols and regular assessments are critical to maintaining the balance between ecological sustainability and human health while sustaining agricultural productivity. Currently, insecticides and fungicides are primarily detected using gas chromatography (GC) techniques. However, some, such as thiamethoxam, are generally determined using high-performance liquid chromatography (HPLC) or liquid chromatography–mass spectrometry (LC–MS) techniques due to their low thermal stability and insufficient volatility.^4,5^ Therefore, direct analysis of these compounds using GC methods is not possible. That means it is challenging to detect residues with different polarities using a single, straightforward extraction method and device. Additionally, traditional chromatographic methods, despite their high sensitivity and accuracy, suffer from several practical limitations, including complex and time-consuming pretreatment steps, expensive instrumentation, limited portability, and incompatibility with real-time or on-site monitoring.^6,7^ Although immunoassays, electrochemical methods, and capillary electrophoresis are common rapid detection techniques, they also suffer from some inherent defects, such as solution instability and limited storage time. Therefore, there is a need to develop fast, simple, and cost-effective analytical methods for noninvasive, rapid, and sensitive detection of pesticides to prevent potential health risks.^8^

Surface-enhanced Raman spectroscopy (SERS) is a sensitive technique that combines Raman spectroscopy with nanotechnology. Weak Raman signals specific to molecules are defined as “molecular fingerprints” and can be enhanced by placing the analyte on nanoscale, rough noble metal (usually silver or gold) surfaces.^9^ Numerous studies in the literature have reported the detection of insecticide and fungicide residues using SERS with different substrates. For some of them, the detection limits (LoD) obtained with this method have been much lower than those achieved with chromatographic methods.^10,11^ The SERS technique offers several advantages, including exceptional sensitivity and selectivity, photostability, single-molecule detection capability, and nondestructive analysis. These features have made SERS a powerful tool in areas such as food safety, environmental detection, and biological analysis. However, practical challenges such as limited quantitative accuracy, poor signal reproducibility, unstable signal behavior, and the complexity of spectral data interpretation, still hinder its widespread implementation.

Two strategies were adopted to address these issues: (1) the development of new SERS substrates that enable the acquisition of high-quality spectra and (2) the integration of machine learning (ML) methods to enable rapid and automated data processing. Under the first strategy, this study designed metal–insulator–metal based honeycomb array (MIM-HCA) surfaces as SERS substrates for pesticide detection. MIM-based hexagonal arrays are highly suitable structures for SERS applications due to their strong surface plasmonic activity and controlled electromagnetic field distribution. The periodic and tightly packed arrangement of the hexagonal geometry contributes to the formation of dense and homogeneous “hot spot” regions,^12^ resulting in a high signal-to-noise ratio in the Raman signal. Due to the plasmonic hybridization between the metal–dielectric-metal layers, the MIM pattern allows for stronger tailoring of the local surface plasmon resonance (LSPR).^13^ The hexagonal lattice morphology provides sensors with high elastic stability and mechanical strength because it is widely available, scalable, and easy to fabricate. These technical properties make honeycomb arrays an appropriate SERS substrate in terms of both signal performance and reproducibility. However, the increase in SERS performance and data reproducibility has inevitably led to an increase in spectral data volume, making data processing more complex and time-consuming. Overlapping signals from different molecules also make it difficult to understand and sort data. Therefore, the rapid and accurate analysis of large data sets is now a crucial need. In this study, a Deep Feedforward (DFF) architecture was used to address this problem. The DFF model demonstrated high performance in separating pesticide signals due to its effective learning ability for the high-dimensional and complex structure of SERS spectra. The nonlinear activation functions between layers are fully accurate in classification and quantification tasks because they can detect even slight differences in spectral vibrations. The simple structure of the model shortened training time and reduced the risk of overfitting. These results demonstrate that the DFF approach can make reliable generalizations in SERS-based analyses even with limited data and can hierarchically learn spectral features. A cucumber matrix was used as a model food to evaluate metalaxyl (MTX), boscalid (BOS), famoxadone (FMX), cypermethrin (CYP), thiamethoxam (TMX), and etoxazole (ETX) for experimental detection of pesticides. The complex molecular structures of these compounds often result in spectral overlaps and baseline drift, introducing noise into Raman spectra. All spectral data were subjected to detailed preprocessing, including Savitzky–Golay filtering, polynomial-based correction, and normalization, rather than simply smoothing. This preprocessing approach effectively minimized noise and baseline shifts, improving both qualitative identification and quantitative prediction performance. The study evaluated six pesticides, comprising three fungicides (MTX, BOS, FMX) and three insecticides (ETX, TMX, CYP), all of which were injected into the cucumber matrix to mimic agricultural conditions. The resulting spectral data were evaluated using both SERS analyses and AI-based data processing models.

## 2 Materials and Methods

### 2.1 Fabrication of MIM-Honeycomb SERS Substrates

In the production of honeycomb arrays, a 10 nm titanium (Ti) adhesion layer and a 100 nm Al layer was initially coated onto the prepared Si substrates via the Torr evaporation technique. ∼150 nm SiN_*x*_ film desired to be deposited on the metallic surface as an insulating layer via PECVD. Subsequently, a CSAR 6200.09 positive resist, appropriate for EBL, was spin-coated to obtain ∼360 nm thickness, and the honeycomb configuration was delineated using an electron beam at 280 μC/cm^2^. After patterning, 10 nm of Ti and 120 nm of Au top metal layers were deposited utilizing a secondary Torr evaporation method. This was followed by a lift-off procedure using the CSAR remover solution (AR 600-71) to obtain a uniform hexagon array. The morphological and optical characterization of the synthesized nanostructures was conducted utilizing different approaches. Structural characterization of the fabricated metasurface was conducted using scanning SEM to evaluate pattern fidelity, periodicity, and large-area uniformity. High-magnification SEM images were used to extract the geometric parameters of the hexagonal nanostructures, including feature size and lattice pitch. Atomic force microscopy (AFM) (Nanomagnetic Instruments–hpAFM) measurements were performed to assess surface topography, height distribution, and phase contrast of the nanostructured surface. Energy-dispersive X-ray spectroscopy (EDS) integrated with SEM (Oxford X_Max- JEOL JIB-4601F MultiBeam FIB-SEM) was employed to determine the elemental composition and distribution within the particles, confirming the fabrication fidelity and the realization of the designed Au HCA on the vertical MIM stack. The thickness of the dielectric layer was determined by spectroscopic ellipsometry (1- J. A. Woollam Co. M2000 Ellipsometer) and found to be ∼166 nm. Optical characterization was conducted using a custom-built reflection micro spectroscopy setup. Reflection spectra were acquired to examine the wavelength-dependent optical response of the metasurface under different refractive index environments relevant to SERS measurements. The SERS performance of the metasurface was evaluated using Rhodamine 6G (R6G) as a Raman reporter molecule. Representative SERS spectra were collected to demonstrate the signal enhancement capability of the fabricated substrate. Multilevel variance analysis of the SERS response was conducted using MTX as a representative analyte to evaluate spatial and fabrication-related signal variability. SERS measurements were performed at a Metalaxyl concentration of 10^–5^ M with a laser power of 100 mW, an integration time of 1 s, and 30 accumulations. To assess spot-to-spot variability, SERS spectra were acquired from 10 distinct spatial locations on a single chip for each independently fabricated batch. Three fabrication batches produced on different dates were analyzed. Batch-to-batch variability was quantified by calculating the RSD of the ensemble-averaged SERS intensities obtained from the three batches. Ensemble averaging was performed hierarchically by first averaging the intensities across measurement spots within each chip, followed by averaging across batches. Chip-to-chip variability was evaluated within Batch 3 by comparing two independently fabricated chips, each measured at 10 spatial locations. All variance metrics were calculated from analyte-containing samples to reflect practical sensing conditions.

### 2.2 Sample Collection, Preparation, GC/MS Validation

Among the pesticides used in this study, MTX, BOS, and FMX are fungicides, while CYP, ETX, and TMX are insecticides. All analytical-grade standards listed in Table 1 were obtained from Sigma-Aldrich (St. Louis, MO, USA). Solvent selection was made for each pesticide, considering the solubility profiles defined in the literature. Acetonitrile was predominantly used for fungicides, while methanol was preferred for insecticides. Stock solutions were prepared by dissolving each pesticide separately in the appropriate solvent and then diluting them in logarithmic concentration ranges (from 10^–4^ M to 10^–9^ M) using the serial dilution method.

**Table 1:** List and Classification of Pesticides Used in This Study (Adopted from the previous study,[Bibr ref14] 2025)

pesticide	chemical name	application	chemical class	CAS no
MTX	*N*-(2,6-Dimethylphenyl)-*N*-(methoxyacetyl)-dl-alanine methyl ester	fungicide	acyl alanine	57837-19-1
TMX	3-(2-chloro-5-thiazolylmethyl) tetrahydro-5-methyl-*N*-nitro-4*H*-1,3,5-oxadiazin-4-imin	insecticide	neonicotinoid	153719-23-4
CYP	[cyano-(3-phenoxy phenyl)methyl]3-(2,2-dichloroethenyl)-2,2-dimethylcyclopropane-1-carboxylate	insecticide	pyrethroid	52315-07-8
ETX	(RS)-5-*tert*-butyl-2-[2-(2,6-difluorophenyl)-4,5-dihydro-1,3-oxazol-4-yl] phenetole 4-(4-*tert*-butyl-2-ethoxyphenyl)-2-(2,6-difluorophenyl)-4,5-dihydrooxazole	acaricide	diphenyl oxazolone	153233-91-1
FMX	3-anilino-5-methyl-5-(4-phenoxy phenyl) oxazolidine-2,4-dione	fungicide	oxazolidinedione	131807-57-3
BOS	2-chloro-*N*-(4′-chlorobiphenyl-2-yl)-nicotinamide	fungicide	nicotinamide	188425-85-6

The sample preparation protocol for GC and LC analyses was applied as follows: first, solid samples were cut into small pieces and homogenized using a food chopper. 5.0 ± 0.1 g of the homogenized samples were transferred to 50 mL centrifuge tubes, and 10 mL of acetonitrile was added. For LC–MS/MS quantification, when internal standardization was required, 50 μL of a 20 ppb Metolachlor-D_6_ solution was added. The tubes were sealed and vortexed for approximately 3 min. Then, 0.5 g of anhydrous sodium acetate (NaOAc) was introduced and vortexed for an additional 3 min to enhance extraction efficiency. Finally, the samples were centrifuged at 4000 rpm for 5 min to achieve phase separation.

The supernatant was subjected to identical cleanup procedures for both GC and LC analyses, with only the reconstitution stage differing between them. For GC analysis, 8 mL of the upper phase was transferred to a 10 mL centrifuge tube containing 2 g of MgSO_4_ and 0.15 g of PSA (Primary Secondary Amine). The tube was vortexed and centrifuged again, and then the supernatant was transferred to a 15 mL glass tube. The extract was evaporated to dryness at 50 °C under a gentle nitrogen stream (≈5 psi), and the residue was directly dissolved in 1 mL of hexane before being transferred into GC vials. The procedure followed for LC analysis was similar; the supernatant was cleaned in the same way, but the residue after evaporation was dissolved in 1 mL of a 50:50 mixture of mobile phases A and B and transferred to LC vials. In this way, both reference solutions and extracts obtained from real food matrices were made suitable for Raman and chromatographic analyses.

### 2.3 Automated Spectral Preprocessing via RamanPlot GUI

Raman measurements were performed using a Raman spectroscopy system equipped with a 785 nm laser source, which was also employed in a previously published study by this group.^38^ All spectral measurements were taken under the same parameters, using a 40× objective with a power of 9 μW. At least 10 spectral measurements were made for each real sample and reference pesticide, and 5 measurements were made for naturally contaminated but unspiked samples. Twenty-five spectral acquisitions were performed for each spectrum, with a 2 s integration time.

The initial phase of data analysis is preprocessing, which seeks to enhance peak visibility by eliminating background noise from the spectra. Preprocessing was conducted utilizing the Python-based RamanSPy library, and all spectra underwent an automatable and reproducible procedure. A unique Raman Spectra Analyzer (RamanPlot) user interface was built to facilitate a more user-friendly and intuitive preprocessing experience. This interface enables the simultaneous loading and automatic processing of several spectra using established pipeline parameters, preparing them for machine learning applications. Upon selecting data files in the interface, the user can modify parameters including crop range, baseline correction technique, denoising method, filtering window length, and polynomial degree. Moreover, peak picking can be enabled, allowing the user to precisely specify threshold parameters (such as prominence, minimum width, minimum height, and minimum distance) for peak identification. The lower half of the interface provides multiple options for exporting processed or raw data, identified peaks, and individual spectra. This not only accelerates the preprocessing phase but also guarantees a consistent and reproducible data processing workflow across all concentration series. The provided snapshot (Figure S5) displays the UI of the Raman Spectra Analyzer. The left side presents the file selection area (Data File 1, Data File 2) and the configuration settings (Crop Start/End, Baseline Method, Denoise Method, Window Length, Polyorder, etc.). The user can provide detailed parameters for peak detection, including prominence, minimum width, minimum height, and minimum distance, enabling the testing of various thresholds during analysis. The preprocessing pipeline employed the Whitaker–Hayes despiking technique to eliminate narrow, high-intensity noise signals caused by cosmic rays. Baseline correction was subsequently executed utilizing the ARPLS (Asymmetrically Reweighted Penalized Least Squares) approach to maintain peaks and normalize nonpeak areas. Savitzky–Golay filtering (window length: 8, polynomial degree: 2) was employed to hold the position and morphology of Raman peaks while attenuating high-frequency noise. All replicated spectra were processed in batches utilizing the identical workflow. Following preprocessing, to mitigate variability caused by temporal aberrations such as heterogeneous sample distribution, sample desiccation, and other variables, spectra were normalized against a fixed experimentally determined background peak (∼1005, 1160, or 1265 cm^–1^). This facilitated dependable comparisons between spectra acquired at varying concentrations. The averaged processed replicate spectra were shown, and calibration curves for typical peak heights on a logarithmic concentration scale were fitted using a logistic function. Detection limits were determined using the calibration curves utilizing the formula [*x* + (3.33 × σ)].^15^ *x* is the highest blank normalized intensity acquired near the characteristic peak (within a 10 cm^–1^ interval), whereas σ signifies the standard deviation at this location. All statistical analysis and LOD calculations were done, and all spectral data were averaged, processed, and visualized using OriginPro 2025 software.

### 2.4 Machine Learning-Based Classification

The pesticide spectra obtained by Raman spectroscopy have been divided into four different data sets for use in classification and quantification studies. This data was processed using the RamanPlot GUI application. Each spectrum consists of 3562 Raman shift points (cm^–1^). The first data set contains only pure pesticide spectrum and consists of six classes (BOS, CYP, ETX, FMX, MTX, TMX). The second data set includes both pure variants and variants mixed with cucumber matrix for each pesticide, thus creating a 12-class structure. The third data set is defined as concentration levels of −4, −7, and −9 units. The final data set contains six different concentration levels ranging from 10^–4^ to^–9^. Only spectral-shift augmentation was applied to the classification data sets. Each training example was randomly shifted ±4 cm^–1^ along the Raman axis, resampled using linear interpolation, and two additional copies were created for each original spectrum, tripling the training data. This made the model more robust against small wavenumber deviations. No augmentation was applied to the quantification data sets. Two main models were established. The first is a classification model. This model was designed as a fully connected (feed-forward) structure with an attention mechanism. Batch normalization was applied, and the importance of weight (mask) for each Raman shift was learned to highlight information-rich regions. 1–2 hidden layers (5–50 neurons, LeakyReLU activation, L2 = 10^–4^) were applied. Dropout was used in the range of 0–0.2 to prevent overfitting. The output was obtained using a multiclass output layer with Softmax activation. The model was trained with the Adam optimizer (learning rate 10^–2^–3 × 10^–4^) and categorical cross-entropy loss. An early stopping criterion (patience = 8) was applied, and hyperparameters were determined using Bayesian optimization (Karas Tuner). In the second model, a dense neural network incorporating residual connections and Squeeze–Excitation (SE) blocks were developed for concentration estimation. Raman spectra with 3562 inputs and σ = 0.01 Gaussian noise were used again. Initial dense blocks were set up using 256–1024 neurons, ReLU activation, and dropout (0.2–0.5). Residual blocks were applied with 2–5 blocks, each with bottleneck compression (25–75%), batch normalization, dropout, and residual skip connections. Channel weighting was performed at a rate of *r* = 8, and the output layer operated with 3 or 6 neurons (concentration levels) with Softmax activation. The model was trained with Adam optimization (learning rate 10^–3^–5 × 10^–5^) and sparse categorical cross-entropy loss, with early stopping up to 50 epochs (up to 80 epochs depending on convergence behavior). Both models were trained on the TensorFlow/Keras 2.19 platform, on an NVIDIA T4 GPU. Seed = 42 was used for randomness control. Hyperparameter optimization was carried out using Keras Tuner (v3.10.0 and v3.12.11). For each trial, the model achieving the highest validation performance was selected by restoring the weights from the epoch corresponding to the maximum validation metric. The data set was first split into training, validation, and test sets using a fixed ratio of 70–15–15%, respectively. To strictly prevent data leakage, all data augmentation procedures were applied only after this initial train/validation/test split and were restricted exclusively to the training set. Augmented spectra were generated solely from original spectra assigned to the training subset. No augmented versions of any spectrum were introduced into the validation or test sets. Consequently, the validation and test sets consist entirely of original, unaugmented spectra.

## 3 Results and Discussion

### 3.1 MIM-Honeycomb Substrate Performance

In this study, hexagonal honeycomb-patterned MIM structures were fabricated using lift-off-based electron beam lithography (EBL). Our patented multilayer architecture (EP Patent EP4486200A1) consisting of aluminum (Al)–silicon nitride (SiN)–gold (Au) layers provided strong plasmonic confinement and increased electromagnetic field intensity due to the optical resonances offered by the metal–dielectric–metal stack (Figure 1a). Thus, the Raman scattering intensity increased, and the reliability of the signals obtained from the analyte molecules improved. MIM-based honeycomb structures offer important advantages in terms of selectivity and sensitivity for pesticide detection due to their extended spectral tunability and low absorption losses. The resonance of honeycomb MIM structures around ∼800 nm strongly overlaps with the 785 nm laser used, and the 400–1800 cm^–1^ shift range of Raman scattering also corresponds to this resonance region. This spectral alignment has effectively enhanced the SERS signal by increasing the electric field intensity.

**Figure 1:**
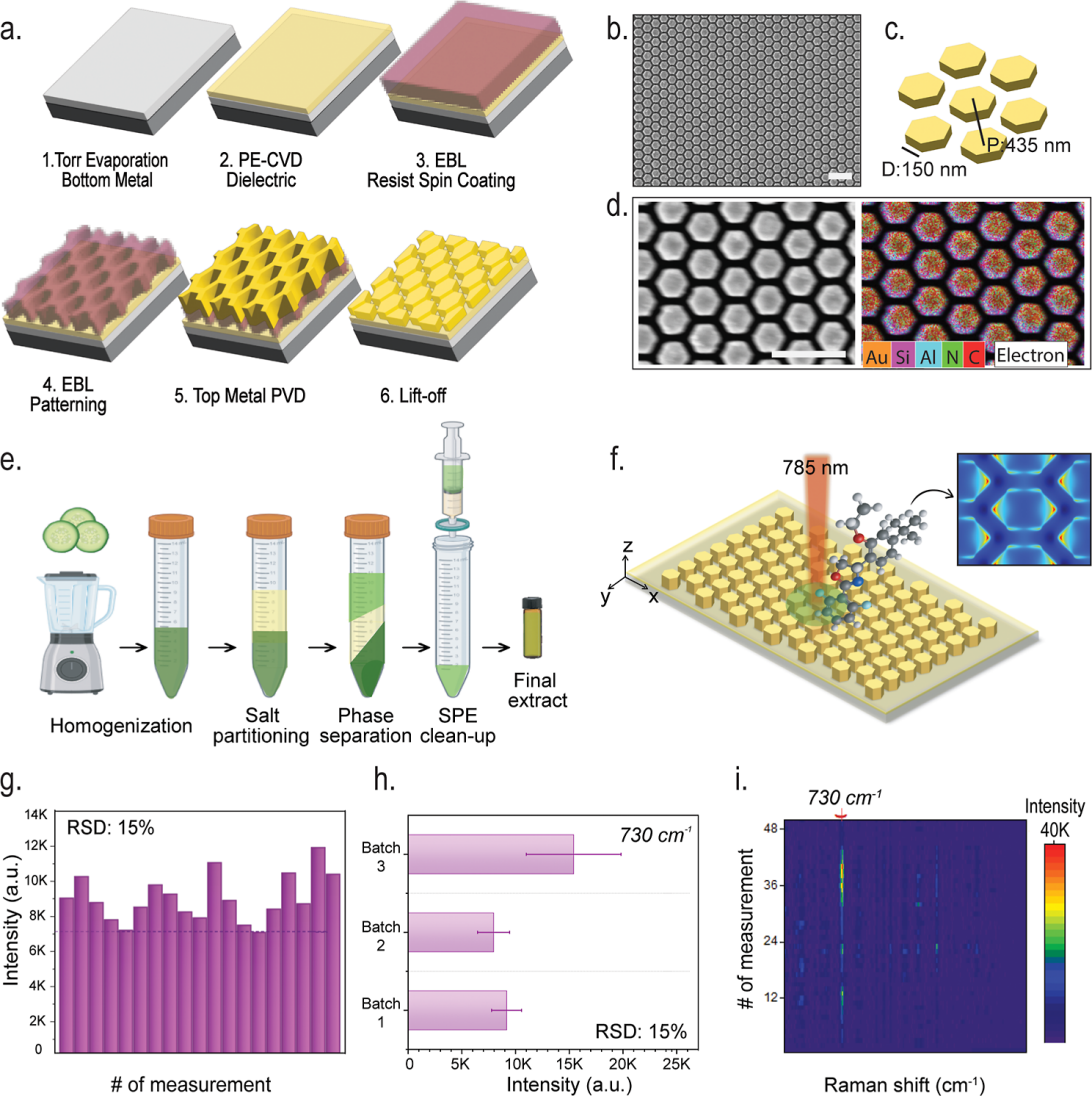
Fabrication process and characterization of a honeycomb array, along with its application in QuEChERS-based pesticide analysis. (a) Substrate fabrication steps: (1) Coating the bottom metal layer via plasma-enhanced chemical vapor deposition (PECVD, (2) depositing the dielectric layer, (3) patterning the CSAR e-beam resist using EBL, (4) exposure followed by development, (5) coating the top metal layer through PVD, and (6) performing the lift-off process. (b) SEM image shows that the hexagonal structures are very regular (scale: 1 μm). (c) Main parameters that construct the 1-unit cell of hexagonal array. (d) The SEM image (left) and the EDS elemental density map (right) of the identical location are presented. The map on the right verifies that the elements Au, Si, Al, N, and C are distributed as intended within the surface design. (e) A diagram showing how to extract pesticides from cucumbers using the QuEChERS method: homogenization, extraction with acetonitrile, phase separation using QuEChERS salts, centrifugation, dispersive SPE cleaning, and making the final extract. (f) Schematic representation of Raman scattering utilizing a 785 nm laser beam focused on hexagonal structures arranged in a honeycomb array. (g–i) illustrates the average SERS intensities of 730 Raman shifts obtained on a blank cucumber matrix and their statistical fluctuations; also, relative standard deviation values calculated from 15 different measurement points are presented; a comparison of RSDs across three different production batches is made, and a heat map of 48 spectra obtained from blank cucumber matrix measurements is presented.

The surface architecture is constructed on a dielectric interlayer between two metal layers; the upper metal layer consists of a hexagonal gold nanoantenna. They demonstrated fine-precision patterning and structural consistency by exhibiting a regular hexagonal arrangement with homogeneous periodicity. As shown in Figure 1b, the structures have been produced uniformly. Regular hexagonal nanostructures with a 435 nm period and 150 nm edge length were precisely defined, while 60 μm × 60 μm areas were optimized as active SERS regions to maximize plasmonic coupling and signal enhancement^16^ (Figure 1c). Elemental mapping verifies the expected presence of Si, Al, Ti, and Au within the HCA regions, with negligible contamination. Trace amounts of C and O originate from residual CSAR resist following lift-off, while S and Ag were detected, albeit in trace amounts (Figure 1d).

Figure 1e illustrates the extraction workflow of the cucumber matrix using the quick, easy, cheap, effective, rugged, and safe (QuEChERS) method. This study systematically evaluated SERS performance not only using reference solutions but also within a real cucumber matrix, which constitutes the primary analytical objective. Considering matrix effects is essential, as agricultural products contain complex organic components that can introduce background signals and interfere with analyte detection. QuEChERS extraction enabled the matrix-derived background to be introduced in a controlled and reproducible manner, ensuring that matrix contributions were recorded as characteristic spectral features rather than random noise. This approach minimizes the risk of false-positive or false-negative interpretations in Raman measurements. Blank cucumber matrix samples which are defined as pesticide-free cucumbers processed using the same extraction and measurement protocol as spiked samples and they were measured on blank substrates to evaluate matrix-induced variability.

SERS measurements obtained from blank cucumber matrix on blank substrates exhibited acceptable reproducibility, with relative standard deviation (RSD) values remaining below 15% across three independently fabricated substrate batches. Although signal intensities varied across various positions on individual substrates, such positional variability is an intrinsic feature of SERS, primarily arising from stochastic laser–hotspot coupling. This effect is further amplified in analyte-containing measurements due to additional contributions from surface adsorption dynamics and molecular orientation in the liquid phase. To obtain representative ensemble responses and mitigate local variability, spectra were acquired from multiple randomly selected positions on each substrate and average. In matrix-spotting Raman measurements, a characteristic acetonitrile peak was consistently observed and used as a spectral reference. Specifically, spectra collected from at least 10 randomly selected positions per substrate across three different substrates were preprocessed and analyzed (Figure 1g,h). The resulting RSD values, typically around 15%, confirm that the HCA-MIM metasurface exhibits acceptable spatial homogeneity, consistent with reproducibility benchmarks commonly reported for SERS-based platforms.^17^ Spatial signal uniformity was further validated by heat-map analysis, which demonstrates consistent spectral behavior across a total of at least 48 spectra collected from three independent fabrication batches (Figure 1i). In addition, a multilevel variance analysis was performed using MTX as a representative pesticide at a practically relevant concentration (10^–5^ M). This analysis decoupled contributions from spot-to-spot heterogeneity, chip-to-chip variability, and batch-to-batch fabrication differences, allowing a systematic assessment of the robustness of both the fabrication process and the SERS response under identical measurement conditions (Figure S10). Reusability tests were conducted using sequential solvent cleaning (acetone–methanol–ethanol); however, residual spectral signatures from previously measured analytes persisted, indicating strong analyte–surface adhesion and a memory effect on the plasmonic surface. This behavior is consistent with known surface modification and interfacial interaction principles, where nanostructured plasmonic substrates promote strong adsorption that enhances SERS signals but limits complete analyte removal.^18^ As a result, repeated use was found to compromise analytical fidelity, and the platform is therefore intentionally positioned as a single-use, high-stability SERS substrate. Long-term stability tests (Figure S11) confirm that the substrate maintains its plasmonic activity and spectral integrity over several days without signal attenuation. Finally, the introduction of the cucumber matrix resulted in a systematic and reproducible background contribution that was recorded prior to each analyte measurement and treated as a characteristic matrix response rather than random noise. For all pesticide analyses, matrix and background spectra were acquired in advance and used as references for subsequent comparison and detailed evaluation of pesticide-specific Raman signatures.

### 3.2 Raman Scattering of Pesticide Residues from Cucumber Samples

Fungicides are chemicals used to control fungal and fungal-like pathogens that cause economic losses in plants. One of these is MTX, a systemic fungicide used to control diseases caused by oomycetes. MTX penetrates plant tissues and inhibits RNA polymerase enzymes, thereby halting RNA and protein synthesis in fungal cells and preventing spore germination and the spread of infection. It is applied to vegetables, fruits, field crops, and ornamentals through seed treatment, foliar spraying, or soil incorporation. Owing to its broad-spectrum activity and systemic mobility, MTX has become an essential component of modern agricultural practices. However, excessive or uncontrolled use may lead to residue accumulation in the environment, pollution of water resources, and adverse effects on nontarget organisms.^19^ Therefore, the rationale use of MTX at the appropriate doses and intervals is critical for maintaining both effective disease control and environmental sustainability. Figure 2 shows the SERS findings for the quantification of MTX in a cucumber matrix in detail. Figure 2a shows the normalized Raman spectra obtained at different concentrations (10^–4^–10^–9^ M). The characteristic Raman bands of each pesticide, along with their corresponding vibrational mode assignments, are taken from the same reference work cited in the literature.^14,20^ Bands at 438, 471, 526, and 545 cm^–1^ are associated with low-frequency skeletal vibrations and ring deformations. A prominent band at 568 cm^–1^ indicates skeletal ring deformations. The vibration in the 765 cm^–1^ region corresponds to aromatic ring-bending modes, while the band observed at 822 cm^–1^ originates from a pseudoscopic C–O–N stretching vibration. The signal at 962 cm^–1^ is attributed to trigonal ring breathing deformation, while the band at 1132 cm^–1^ represents the C–C stretching vibration. These characteristic peaks confirm the Raman-active vibrations of the aromatic and heteroatom-containing groups in the structure of MTX and serve as a fingerprint that can be used for structural identification of the molecule. The absence of these bands in blank cucumber matrix data suggests that the pesticide can be selectively isolated in the complex matrix. Figure 2b shows a zoom-in of the MTX ∼962 cm^–1^ band as it changes with increasing concentration. The results show that the MTX signal differs from the low-level blank cucumber matrix spectrum and that the signal intensity increases as the concentration increases, confirming the platform’s sensitivity to pesticide detection. Figure 2c concludes the quantitative assessment, showing the calibration curve and the LoD derived from the ∼962 cm^–1^ band. The determined LoD is 4.8 × 10^–7^ M, indicating nanomolar sensitivity for MTX analysis. It is generally known that a linear approximation is applied to calibration curves in SERS publications. However, for these types of sensors, the area or intensity values in the Raman spectra do not always increase linearly with increasing concentration. Signal decay is faster at low concentrations, decreases on active surfaces, and slows at high concentrations to form a plateau due to saturation at hot spots. Currently, most quantitative analyses of pesticide residues on fruits and vegetables using SERS detection technology rely on the linear quantification of a single characteristic peak of pesticides.^21^ However, during the actual detection process, the Raman characteristic peak of pesticides tends to shift slightly because of continuous appearance, such as noise, on the instrumentation and characteristics. Therefore, quantitative performance of the method will be affected in practice. This accuracy can be improved through multivariate and nonlinear distribution.^22^ Accordingly, the system response is better described by a sigmoidal (logistic) function rather than by a linear or normally distributed model. Logistic fitting captures both the low-concentration detection regime and the signal saturation observed at higher concentrations, thereby extending the sensor’s effective dynamic and practical operating range. Signal intensities were found to vary across various positions on the substrate, an inherent characteristic of SERS measurements arising from stochastic laser–hotspot interactions as well as analyte adsorption dynamics and solubility effects at the surface. To account for this positional variability, spectra were collected from multiple regions of each substrate and subsequently normalized. Normalization to a fixed reference peak enables meaningful comparison between spectra by reducing signal fluctuations associated with local SERS enhancement inhomogeneities, ultimately yielding more reliable and reproducible results. Furthermore, the determined MTX LoD value (∼0.1 ppm) was below the 0.5 ppm MRL (Maximum Residue Limits) limit reported for cucumbers in the EFSA 2025 reports (https://commission.europa.eu/index_en), demonstrating that the developed system can detect MTX below legal limits.

**Figure 2:**
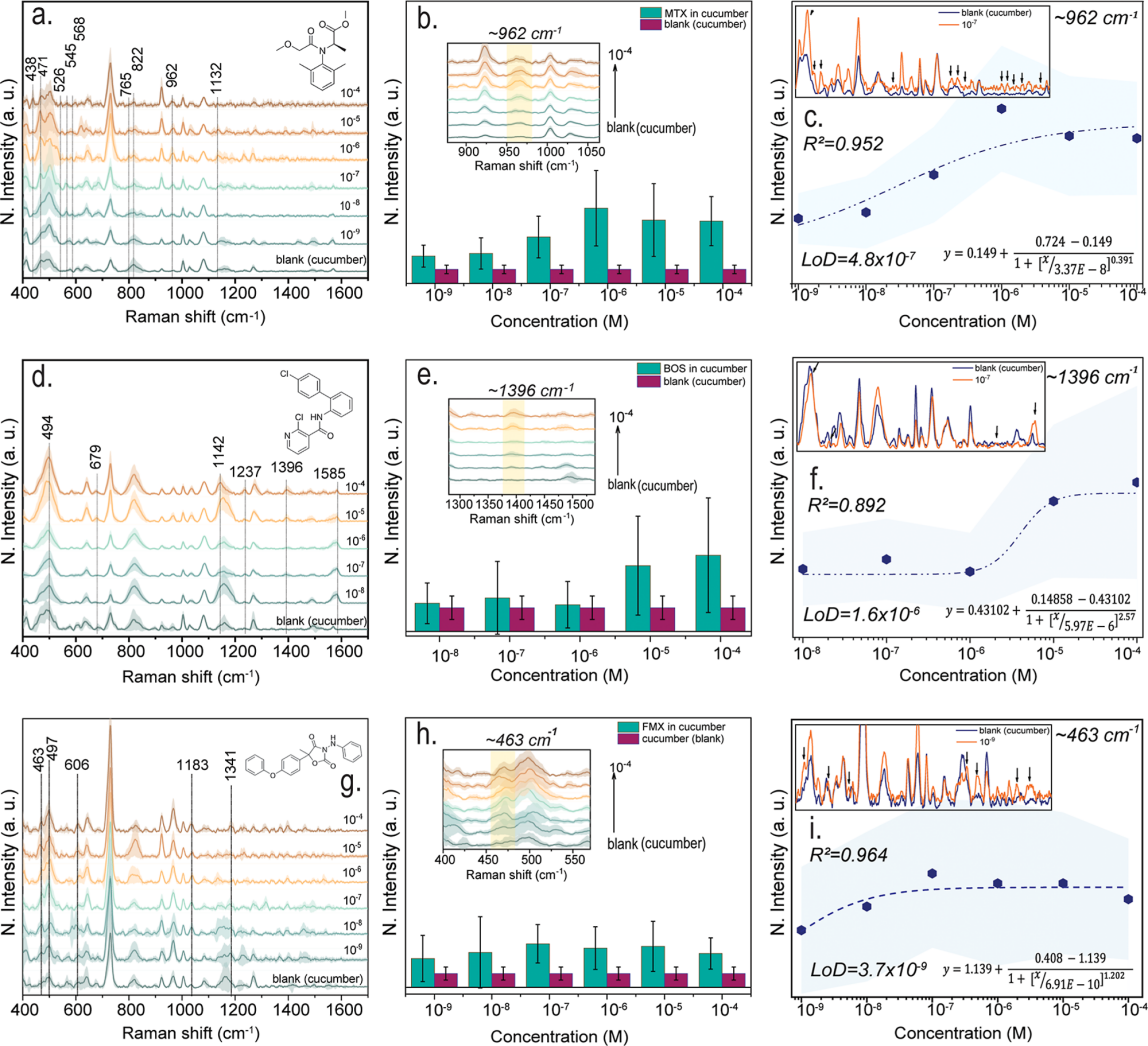
SERS measurements, analysis of characteristic bands, and calibration curves for fungicides; MTX, BOS, and FMX. (a,d,,g) Normalized SERS spectra of MTX, BOS, and FMX recorded in a cucumber matrix at varying concentrations (10^–4^–10^–9^ M for MTX and FMX, and 10^–4^–10^–8^ M for BOS). All SERS measurements were performed using a 785 nm excitation laser with a laser power of 100 mW, an integration time of 2 s, and 20 accumulations per spectrum. For each concentration, a minimum of 10 spectra were collected from different locations on the substrate to account for spatial variability. A blank matrix spectrum obtained by depositing pure cucumber matrix onto the substrate is shown for comparison. The molecular structures of the analytes are included in the corresponding panels. Dashed arrows indicate the characteristic Raman bands of each pesticide. (b,e,h) Enlarged views of the diagnostic bands of MTX, BOS, and FMX at 962, 1396, and 463 cm^–1^, respectively, illustrating the variation of mean SERS signal intensity as a function of concentration. Each data point represents the average of at least 10 independent measurements, with error bars indicating the corresponding standard deviations. Control measurements of the blank cucumber matrix are also included. (c,f,i) Calibration curves constructed from the characteristic SERS bands of each pesticide. The experimental data were fitted using a logistic function, as shown in the inset equation. The upper inset compares the blank cucumber matrix spectrum with spectrum acquired at concentrations close to LoD. Based on the logistic fitting, the LoD values were determined to be 4.8 × 10^–7^ M for MTX, 1.6 × 10^–6^ M for BOS, and 3.7 × 10^–9^ M for FMX. The reported *R* ^2^ values were calculated using a linear fit of three consecutive data points in the quasi-linear region.

Figure 2d shows the SERS spectra obtained at different BOS concentrations in the cucumber matrix. In all spectra, skeletal deformation vibrations were observed at 494 cm^–1^, and aromatic ring bending bands were observed at 679 cm^–1^. C–C stretching bands were detected at 1142 cm^–1^, C–N stretching bands at 1237 cm^–1^, C–H bending bands at 1396 cm^–1^, and aromatic C=C stretching bands at 1585 cm^–1^. A gradual decrease in the intensity of these bands was observed as the concentration decreased. When compared with the spectrum of the cucumber matrix, it is seen that the BOS peaks are clearly distinguishable. Figure 2e compares the blank cucumber with samples containing different BOS concentrations. Particular attention is paid to the band around ∼1396 cm^–1^, and Figure 2f shows the calibration curve based on the signal–concentration relationship obtained in this band. The logistic fit applied to the curve resulted in a detection limit (LoD = 1.6 × 10^–6^ M) of approximately 0.5 ppm, which is much lower than the 4 ppm MRL limit set by EFSA for cucumbers. Furthermore, the blank cucumber matrix signal seen in the inset spectra confirms that the peaks specific to BOS are particularly prominent in the 10^–6^ M BOS sample. BOS is a common fungicide belonging to the nicotinamide class and can be found at high residue levels, particularly in fresh fruits and vegetables (cucumbers are one of them). Although its acute toxicity is low, long-term exposure may cause toxic effects on the liver and thyroid. Its endocrine disrupting potential and the tendency of its metabolites to accumulate in the environment require careful monitoring of this pesticide.^23^ Furthermore, the aromatic rings and chlorinated functional groups in their structure contribute to the effective enhancement of Raman signals by providing strong binding to the metal surface.

Another fungicide, FMX, has been shown to inhibit ATP production by blocking the mitochondrial electron transport chain (cytochrome bc_1_ complex) in fungal cells, provides to be effective against oomycete pathogens such as molds. However, for all agricultural activities, genotoxic effects, liver and kidney damage, and endocrine-disrupting potential have also been reported in chronic exposures of FMX.^24^ Furthermore, due to its lipophilic nature, it poses a risk of accumulation in the environment and exhibits high toxicity to aquatic organisms.^25^ Figure 2g shows the concentration-dependent SERS spectra of FMX obtained at different concentrations in cucumber matrix. Characteristic vibrational bands were identified at 463 cm^–1^ (C–Cl bending), 606 cm^–1^ (oxazolidinedione ring vibration), 1183 cm^–1^ (C–N stretching and ring vibration), and 1341 cm^–1^ (aromatic C–C stretching). Compared to the blank cucumber matrix, the intensity differences in these bands presented the detection of FMX fingerprint signals. Furthermore, the observation of distinct peaks even at the lowest concentration of 10^–9^ M demonstrates the high detection capability of the platform. The double carbonyl (C=O) group in the FMX molecular structure contributes to the effective enhancement of Raman signals by providing strong binding to the metal surface.^26^ In Figure 2h, shows the selective change in the 463 cm^–1^ band in response to concentration changes. The calibration curve created based on this band is given in Figure 2i and the calculated detection limit (LoD = 3.7 × 10^–9^ M, ∼0.001 ppm) is well below the 0.2 ppm MRL limit set by EFSA for FMX.

The widespread practice of using commercially available insecticides to eliminate unwanted crawling and flying insects poses a potential risk of container leakage and soil and water pollution, which can cause damage to both target and nontarget species globally. CYP, a synthetic pyrethroid, is a broad-spectrum insecticide used extensively in households. The rapid and precise detection of CYP is necessary due to its extensive use in agricultural applications and its potential for severe human health effects (for instance; muscle weakness, respiratory problems, seizures) when misapplied.^22^ Figure 3 displays the detection and quantification results of CYP in the cucumber matrix using SERS. The characteristic bands specific to CYP were clearly distinguished from the blank samples in the spectra obtained at various concentrations (Figure 3a). Adsorption of the analyte to the surface is essential for effective detection by SERS. The molecular structure of CYP contains a carbonyl (C=O) group that enables it to bond to metal surfaces, thereby facilitating the effective amplification of Raman signals. In particular, the peak at 642 cm^–1^, which is associated with C–Cl stretching and ring deformation, was chosen for quantification because of its concentration-sensitive behavior. Other characteristic bands include 1146 and 1179 cm^–1^ (aromatic C–H in-plane bending), 1209, 1264, and 1280 cm^–1^ (aromatic C–H in-plane bending and C–C/C–N stretching vibrations, with relative contributions depending on the molecular structure and adsorption geometry on the Au surface). The intensity of 642 cm^–1^ band in Figure 3b increased systematically as the concentration increased and showed a statistically notable difference from the blank cucumber matrix measurements. The logistic function was employed to fit the calibration curve in Figure 3c, and the LoD was determined as 6.34 × 10^–7^ M (∼0.2 ppm) and found to be compatible with MRL limits established for cucumber.

**Figure 3:**
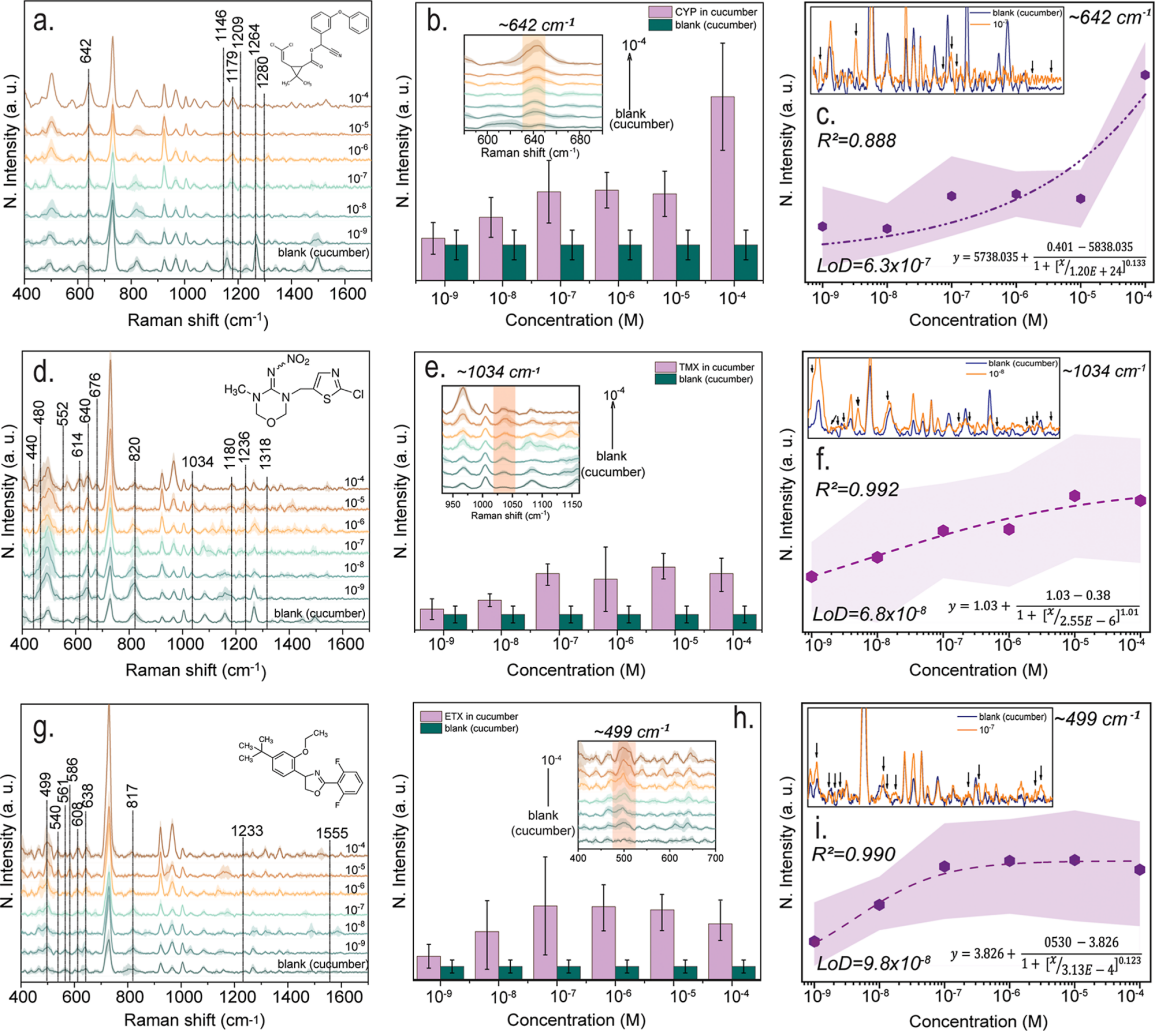
SERS measurements, analysis of characteristic bands, and calibration curves for insecticides; CYP, TMX, and ETX. (a,d,g) Normalized SERS spectra of CYP, TMX, and ETX recorded in a cucumber matrix at varying concentrations (10^–4^–10^–9^ M). All SERS measurements were conducted using a 785 nm excitation laser with a laser power of 100 mW, an integration time of 2 s, and 20 accumulations per spectrum. For each concentration, a minimum of 10 spectra were collected from different locations on the substrate to account for spatial variability. A blank spectrum obtained by depositing blank cucumber matrix onto the substrate is shown for comparison. The molecular structures of the analytes are included in the corresponding panels. Dashed arrows indicate the characteristic Raman bands of each pesticide. (b,e,h) Enlarged views of the diagnostic bands of CYP, TMX, and ETX at 642, 1034, and 499 cm^–1^, respectively, illustrating the variation in mean SERS signal intensity as a function of concentration. Each data point represents the average of at least 10 independent measurements, with error bars indicating the corresponding standard deviations. Control measurements of the blank cucumber matrix are also included. (c,f,i) Calibration curves constructed from the characteristic SERS bands of each pesticide. The experimental data were fitted using a logistic function, as shown in the inset equation. The inset compares the blank spectrum with spectrum acquired at concentrations close to LoD. Based on the logistic fitting, the LoD values were determined to be 6.34 × 10^–7^ M for CYP, 6.8 × 10^–8^ M for TMX, and 9.8 × 10^–8^ M for ETX. The reported *R* ^2^ values were calculated using a linear fit of three consecutive data points in the quasi-linear region.

TMX is a neonicotinoid insecticide that is manufactured from nicotine. It is employed extensively in agricultural environments to manage a variety of pests, such as thrips, leafhoppers, whiteflies, lice, aphids, weeds, and pathogens. Due to its high-water solubility, there is a high likelihood that TMX will be discharged from agricultural fields into nearby water bodies. This contamination of water will have a detrimental effect on aquatic ecosystems, disrupt food chains, and pose a risk to fish and other aquatic organisms.^27^ Additionally, the potential for TMX residues to accumulate in water and soil for an extended period is high due to their ability to persist in the environment for extended periods. This accumulation can result in long-term ecological consequences. In this situation, the detection of neonicotinoid insecticides, such as TMX, is crucial for protecting human health and maintaining environmental balance. For this reason, the study also focused on the detection and quantification of TMX. Figure 3d shows the normalized SERS spectra of TMX at different concentrations ranging from 10^–4^ M to 10^–9^ M in the cucumber matrix. At the bottom, the blank cucumber matrix spectrum is shown. The chemical structure in the upper corner shows the structure of TMX, which contains a nitramine and thiazolidine ring. Distinctive Raman peaks specific to TMX are observed to be around 440, 490, 614, 640, 676, 820, 1034, 1180, 1236, and 1318 cm^–1^. These peaks correspond to NO_2_, C–N, C–Cl, and triazole ring vibrations present in the molecular structure of TMX. The region between 950–1150 cm^–1^ has been enlarged to show the distinctive ∼1034 cm^–1^ band of TMX in detail (Figure 3e). This peak originates from C–S or N–O vibrations in the molecular structure of TMX. The apparent shift of the ∼1034 cm^–1^ Raman band within the 1030–1036 cm^–1^ range observed at certain concentrations can be attributed to intrinsic chemical and instrumental variations inherent to SERS measurements. These variations likely arise from reduced signal-to-noise ratios affecting peak center determination, adsorption-related effects, and local chemical or thermal contributions. Importantly, this shift does not alter the overall concentration-dependent trend. For this reason, the band was denoted as 1034 cm^–1^, and its intensity was extracted using a narrow integration window (1030–1036 cm^–1^). By accounting for matrix-related background contributions and spatial variations in SERS enhancement, the influence of minor peak center shifts was reduced, allowing concentration-dependent calibration to be constructed. This panel shows the relationship between the integrated intensity of the ∼1034 cm^–1^ band and TMX concentration. The data were fitted using a logistic function to capture the nonlinear response observed across the investigated concentration range, including signal saturation at higher concentrations. Based on this analysis, the limit of detection was estimated as 6.8 × 10^–8^ M. While the calibration shows increased scatter at low concentrations, the inset indicates a reasonable correlation in this regime (*R* ^2^ = 0.930). The representative spectrum shown above illustrates the spectral difference between the blank and the 10^–8^ M TMX sample, with arrowheads marking the TMX-related features detectable near the detection limit (Figure 3f).

Another fungicide we interested in is ETX, that functions as a contact-killing acaricide, effectively inhibiting the embryonic development and molting processes of mites. Reports indicate that ETX induces toxic effects, including neurotoxicity, genotoxicity, cytotoxicity, infertility, endocrine-disrupting activity, and oxidative stress in various nontarget organisms. The regulation of pesticides is essential, as the accurate administration of dosage is vital for both human and environmental health.^28^ The absence of comprehensive research on ETX in literature emphasizes the importance of a more profound comprehension of its quantification and detection. Here, we present normalized SERS spectra of ETX obtained in a cucumber matrix at concentrations ranging from 10^–4^ M to 10^–9^ M (Figure 3g). Prominent Raman bands in the spectra are located at approximately 499, 540, 561, 586, 608, 638, 817, 1233, and 1555 cm^–1^. These bands correspond to the C–O–C, C–F, and C–H bending and aromatic ring vibrations found in the chemical structure of ETX. The 499 cm^–1^ band is attributed to the C–F stretching vibrational mode of the molecule and has been considered a characteristic fingerprint. The resulting LoD value was calculated as 9.8 × 10^–8^ M, which is found to be similar to the MRL ∼0.01 ppm (Figure 3h).

### 3.3 From Raw Input to ML-Ready Output

The development of artificial intelligence technologies has paved the way for the emergence of advanced methods that enable the extraction of information, detection of patterns, and predictive analysis from complex data sets. ML algorithms demonstrate significant potential in extracting meaningful information from spectral data, paving the way for transformative advancements in real-world applications.^29^ Recent studies have highlighted that SERS imaging and real-matrix analysis are often limited by substrate heterogeneity, fluorescence background, and the challenge of extracting quantitative information from large spectral data sets; AI-assisted analysis tools have been proposed to mitigate these issues.^30^ In a traditional ML process, raw data undergoes a preprocessing stage, followed by the development of a classifier model and the execution of prediction operations. One of the most critical steps in this process is hyperparameter optimization, which enables achieving the highest model performance. Although data cleaning and hyperparameter optimization are often considered independent steps in Raman spectroscopy applications, these two processes are related. This is because data effective preprocessing not only removes noise and baseline distortions but also enhances relevant spectral features, thereby facilitating more efficient learning and improving model robustness.^31^

For Raman spectra to be effectively utilized in ML models, data standardization must be ensured, and confounding variables must be eliminated. At this stage, spike errors caused by cosmic rays, random noise, and baseline shifts, which are frequently seen in spectrum, must be eliminated.^32^ If these steps are neglected, both qualitative and quantitative analyses are adversely affected, and the classification ability of ML models weakens. As shown in Figure 4a raw Raman spectra typically contains intense fluorescence background, experimental baseline drift, and random noise; therefore, direct interpretation is often impossible. For this reason, all obtained spectra underwent a comprehensive preprocessing process. First, baseline correction was applied to reduce background signals and eliminate systematic shifts affecting the spectrum position. To reduce noise, the Savitsky–Golay filtering method was used to smooth the signals,^33^ thereby preventing random noise from interfering with the spectral signal. Furthermore, normalization was performed to reduce variations arising from different measurement conditions, ensuring that all spectra were comparable. Following these steps, the spectra were cleaned of excess fluorescence background, the signal-to-noise ratio was improved, and a repeatable and reliable data set was obtained. The intensity and signal-to-noise ratio of Raman spectra can vary depending on measurement conditions, changes in laser power, integration time, and analyte concentration, leading to noticeable differences in intensity between spectra.

**Figure 4:**
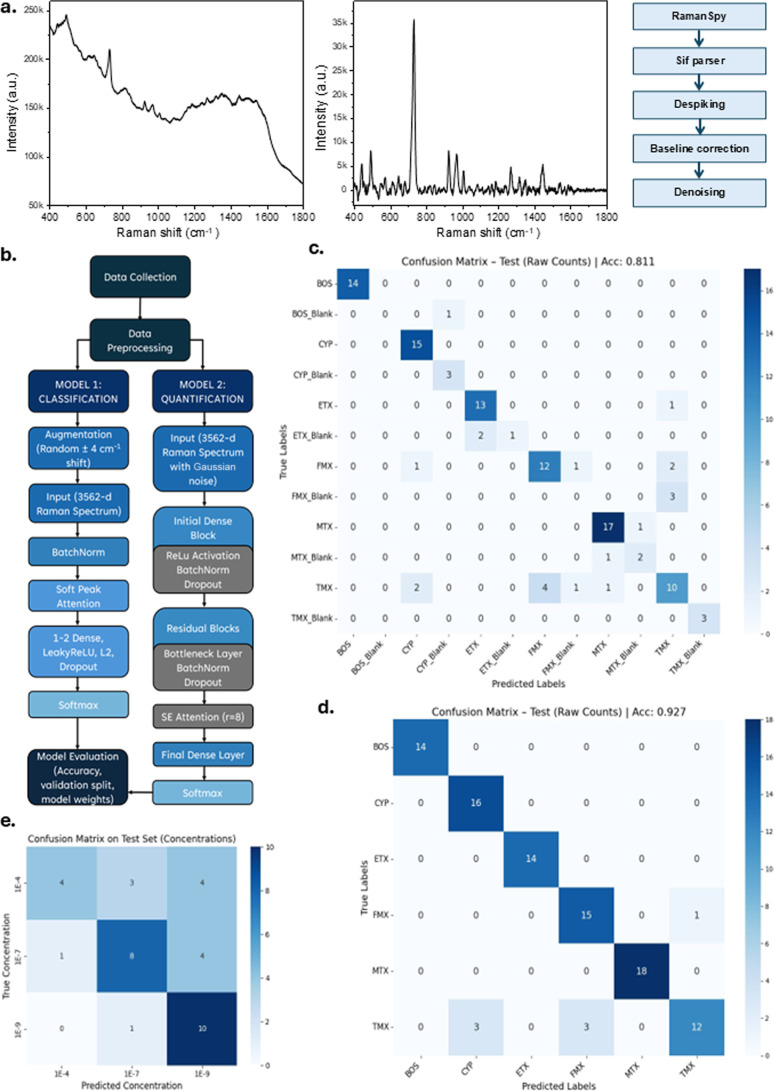
General workflow, model architecture, and summary representation of performance outputs for the pesticide classification and quantification models for the MIM-SERS Platform. (a) The RamanSpy-based data processing pipeline is shown at the top. Raw.sif format Raman spectra were preprocessed by passing through the RamanSpy, sif parser, despiking, baseline correction, and denoising stages, respectively. Graphs representing preprocessed and unprocessed data obtained from TMX are shown. (b) Flowcharts prepared for two different ML models are included: model 1 predicts pesticide types or blank variants as a multiclass classification. model 2 predicts concentration levels (in the range of 10^–4^ M–10^–9^ M). (c) The confusion matrix for model 1 on the test set is provided. This matrix shows that a classification accuracy of 81.1% was achieved across 12 classes (6 pesticides +6 blank variants). (d) Shows the test result performed only with pure pesticide classes, achieving an accuracy rate of 92.7%. (e) Presents the results of quantification performed for three different concentration levels (10^–4^ M, 10^–7^ M, 10^–9^ M).

### 3.4 ML Models for Identification and Quantification of Pesticide Residues in MIM-SERS Platform

Although the MIM-SERS platform can generate a vibrational fingerprint specific to each pesticide residue, the numerous simultaneous components present in cucumber samples can mask these unique signals due to background signals. Furthermore, it is quite difficult to detect small spectral changes in such a complex system manually. Therefore, two different ML algorithms were created to distinguish pesticide fingerprint bands from complex SERS signals. After the spectra were collected, a preprocessing step involving noise reduction, baseline correction, and normalization was applied. The data was then directed to two different models. In model 1 (Classification), data augmentation was applied to the spectra, scaling was performed using batch normalization, and important wavenumber regions were highlighted using the Soft Peak Attention layer. This was followed by one or two hidden layers with LeakyReLU activation, L2 regularization, and dropout. At the output, pesticide classification was performed using a layer with a SoftMax activation function. Independent from model 1, in the model 2 (Quantification), spectra with added Gaussian noise were used as input. This model consists of an initial block with ReLU activation, batch normalization, and dropout, followed by several residual blocks and a Squeeze–Excitation (SE) attention mechanism (*r* = 8). The final layer contains a dense layer with Softmax activation, and pesticide concentrations are estimated. The performance of both models was evaluated based on accuracy, validation, and best model weights (Figure 4b).

Confusion matrices showing the classification performance of the developed ML models on the test data are compared. Figure 4c shows the model trained on a data set using both spiked and pesticide-free matrix variants. In this case, two separate classes were defined for each pesticide, for example, for BOS, BOS_Blank was defined as its blank cucumber matrix. According to the results, although the model could generally distinguish between classes, the accuracy rate dropped to 81.1%. Some TMX_Blank samples were classified as TMX, while some ETX_Blank samples were classified as ETX. This confusion can be attributed to the pesticide bands being obscured by the matrix background, particularly at low concentrations. In contrast, Figure 4d shows the situation where only spectra from the spiked samples were used. In this scenario, the accuracy rate increased to 92.7%, and the pesticide classes were separated. The misclassification rate is lower, and the high values on the diagonal indicate that the model has strong prediction. Since there were no blank signals, the model learned only the spectral features originating from pesticides, and the uncertainty caused by background effects was eliminated.

The spectral differences within blank cucumber samples directly affect model performance. Even if they belong to the same biological matrix, blank samples exhibit different Raman profiles due to variables such as moisture and pigment content. This situation is particularly evident in some blank cucumber samples in the 400–1200 cm^–1^ range, with peaks concentrated around 700 cm^–1^ in some and around 1000 cm^–1^ in others. Even if they belong to the same class (as shown in CYP or BOS), small shifts in peak positions, intensity differences, and baseline changes can be observed (Figures S2 and S3). Using at least 10 spectra for each pesticide and their normalized versions enabled the model to learn both the chemical fingerprint and the variations caused by the background effect. However, since the model makes predictions based on peak position and intensity relationships, the blank class becomes inconsistent in data sets with high intrinsic variation. If all blank samples were grouped under a single class, the model would encounter conflicting peak patterns within that class and would not be able to identify the spectral feature that represents the blank correctly. This situation would lead to an increase in false negative predictions, especially for low-concentration pesticides that are under conditions where the signal is weak.

Including the blank class also increases the number of classes, making it more difficult to distinguish between the matrix-only and the matrix with pesticide signals. At low concentrations, the signal-to-noise ratio decreases, and pesticide bands become indistinguishable from the matrix background, resulting in spiked samples that spectrally approach the blank. This situation led to an increase in misclassifications, particularly in lower concentrations. Furthermore, background variation and peak shifts made the decision boundaries unclear. Therefore, the decrease in accuracy is a natural consequence of a more realistic but more challenging classification problem arising from the addition of the blank class. Training the blank variant specific to each pesticide’s matrix as a separate class has ensured that the model learns not only the pesticide signal but also the characteristic spectral changes resulting from the interaction between the pesticide and its matrix. Thus, the system became sensitive not only to the differentiation between the presence or absence of the pesticide but also to the signal patterns specific to each pesticide’s own matrix, thereby increasing its detection ability on real samples.

Model 2, which was developed to enable the platform to perform quantification, was initially trained across a wide range covering all concentrations (6 different concentrations between 10^–4^ M and 10^–9^ M); then, it was changed to predict between 3 characteristic concentrations (10^–4^ M, 10^–7^ M, and 10^–9^ M) by narrowing the concentration range. This narrowing process reduced spectral similarities between classes, allowing for a clearer observation of the quantification ability of the model. When all concentrations were included, the spectra at intermediate levels (10^–6^ M and 10^–7^ M) were frequently confused with both higher and lower concentrations; this resulted in an overall accuracy of approximately 51% (Figure S4). Furthermore, signal saturation at high concentrations and noise effects at low concentrations limit the prediction performance. With this narrowing, where the concentration range was set to be approximately the highest, medium, and lowest concentrations, the performance of the model was seen to improve. In the new confusion matrix, it was observed that the model predicted with high accuracy (10 correct, 1 incorrect) at the 10^–9^ M level and showed consistency with 8 correct predictions at the 10^–7^ M level. Although some confusion persists at the 10^–4^ M level, the overall accuracy rate has increased to 62.9%, and the average F1-score has reached ∼0.61 (Figure 4e). The fact that the precision values fall within the range of 0.67–0.80 indicates that a significant portion of the samples predicted by the model belong to the correct class. In particular, the recall value of 0.909 for low-concentration samples (10^–9^ M) proves that the model is sensitive at this level. Since the model examines the relative distribution of the pattern rather than just the signal intensity, it learns the shape created by all the peaks in the spectrum collectively. If this pattern is consistent even at low concentrations, the model can classify correctly. This is the advantage of nonlinear feature mapping of ML compared to classical calibration curves. At the nanomolar levels (10^–9^–10^–8^ M), where low signal intensities prevail, it still exhibits over 60% quantification, demonstrating that our platform could be used as a comprehensive package of pesticide analysis for field-deployable applications. However, due to signal variations and limited data, the accuracy of the model varies across concentration ranges. Improvements in the data set and signal enhancement strategies are believed to increase the quantification performance of the model. Moreover, from an application perspective, such class-based quantification is often sufficient for food safety monitoring, where the critical information is whether pesticide residues exceed or fall below regulatory thresholds, rather than their exact concentration values.

Nguyen et al.^39^ developed a paper/GO/e-Au flexible SERS sensor for in situ detection of pesticide in orange juice and on cucumber skin at the subppb level, incorporating ML-assisted data analysis. They used composition for SERS active materials for comparison Selectivity for small molecules diversity of pesticide monitoring Therefore, the model is at risk of overfitting due to the small data set, which consists of 50 total samples (5 per class, with 3 for training and 2 for testing). Furthermore, they used 2–4 hand-selected peaks instead of the entire spectrum introduces both bias and vulnerability to peak shifts/baseline changes. In other study, Wang et al.^49^ demonstrated a highly sophisticated approach focused on SERS imaging and image processing using an Ag@BN nanoparticle-based system, achieving high accuracy classification over a large data set (>5000 spectra) compared to this study. Li et al.,^37^ working with Ag nanocubes in water, reported an accuracy of ∼0.967 on >9000 spectra using portable Raman and ResNet at exceedingly high power (300 mW). Additionally, it was reported that the system was unable to perform quantification. Sun et al.^48^ detected deltamethrin in drinking water using a silver sol-based system, reporting 98% accuracy with a GRU–CNN attention architecture. However, the matrix used was relatively simple and limited to a single target pesticide. Girones et al.^46^ detected pesticides in aqueous solutions on a stainless-steel substrate, achieving 99% accuracy with a 1D-CNN. Evaluating the system with standard solutions, independent of actual matrix effects, is beyond the realm of practical application. Hegde et al.^35^ introduced the multitask, transformer-based SERSFormer-2.0 model, which utilizes SERS data for the simultaneous detection and quantification of multiple pesticides in agricultural products. The model operates with task-specific layers and a shared multihead attention encoder, reporting near-perfect performance. The strong SERS signals obtained with core–shell Au–Ag nanoparticles, along with the analysis of spectral dominance mechanisms, demonstrate the potentials of the method to identify mixed contaminants and contribute to regulatory applications reliably. Compared to these colloidal based detection techniques, our MIM-HCA metasurface demonstrates subppb sensitivity on a reproducible and production-ready platform, enabling not only the detection of presence or absence but also the quantification of target analytes within a real food matrix. The method offers interpretable decision regions through peak-weight analysis and the stability advantage of its robust layered structure, making it more field-integrated. Hotspots on MIM-HCA offer values such as spatial repeatability and proximity to CMOS-compatible production. This enhances field applicability and the perspective of mass production, unlike most “nanoparticle suspension” approaches in the literature.^34^

Our developed MIM-HCA integrated platform achieved LoD around MRL limits and at subppb levels that provides sensitivity compared to ppm-level LoDs reported in some studies. However, its ability to produce stable discrimination even at very low levels such as 10^–9^ M in blank-free sets makes this study stand out, despite the data set size being considerably lower than studies in literature with >2,000, >9,000, or even image-based more than 10^6^ samples (Table 2). This difference inevitably creates pressure on both class imbalance and subgroup classification; it is also one of the most crucial factors limiting accuracy. Although a more comprehensive data set could potentially enhance performance, our methodology demonstrates that even a restricted set of data, which may be collected on-site before testing, can yield satisfactory outcomes in real-world matrices. Of course, it is essential to collect training data that closely resembles the actual data in data-driven methods such as deep learning. The specific application will determine the precise parameters of the actual data and the size of the data set required to represent it accurately. In addition to individual analyte measurements, various dual pesticide combinations such as BOS-MTX, FMX-ETX, MTX-TMX and natural contaminant samples were also investigated on the developed MIM–SERS platform (Figures S6–S8). The partial peak overlaps and spectral interferences observed in these combinations highlight the importance of signal resolution in multiresidue systems. Nevertheless, ability of the platform to maintain characteristic vibration regions even in these complex systems demonstrates the structural stability and spectral accuracy of the system in terms of distinguishing coexisting pesticides. These results indicate that the proposed MIM–SERS platform also provides a basis for future multiresidue detection and analysis of complex matrices.

**Table 2:** Comparison of the Present Work in Literature

study	LOD	substrate	matrix	analyte	acquisition settings	instrument	# of spectra	model	quantification	performance
^35^	0.5 ppm	Au@coreshell	spinach	coumaphos	20 mW laser power	DXR2 Raman spectrometer	>30000	CNN, ReLu,transformer models	yes	accuracy = 0.999; F1 score = 0.992; precision = 0.990; recall = 0.996
		Ag NPs	strawberry	oxamyl						
				carbophenothion						
				thiabendazole						
				phosmet						
^36^	1 ppm	Ag NPs	environmental	thiram	30 mW laser power	portable Raman spectrometer (BWS415–785-H)	-	CNN	NA	accuracy = 1
			contaminants	triadimefon	5 s integration			spectral angle model		loss = 0
				benzimidazole				Cuda and ReLU		
				thiamethoxam						
^37^	0.01 ppm	AgNCs	environmental water	methamidophos	300 mW laser power	portable Raman	>9000	ResNet	NA	accuracy = 0.967
				dimethoate		spectrometer (i-Raman Plus)				
				glufosinate ammoniumethyl						
				para-nitro-phenyl						
				parathion						
				phosmet						
^38^	0.01 ppm	PS sphere array	fruit juice	parathion-methyl	50 mW laser power	confocal Raman	>500	python package sklearnSVM	NA	-
				carbaryl	10 s integration	spectrometer (model inVia, Renishaw)				
				ferbam						
				ziram						
^39^	0.001 ppm	paper/GO/e-Au flexible nanosheets	cucumber skin	tricyclazole	45 mW with a 45° contact angle	MacroRaman	∼50	multiclass classification ML models: LR, k-NN	yes	accuracy = 1
			orange juice			Raman spectrometer (Horiba)		SVM, decision Tree, RF, Naive bayes		loss = 0 quantification accuracy = > 0.5 (for real samples)
^40^	5 ppm	GNPs	beetroot juice	thiram	25–50 mW laser power	fiber optic Raman	>1500	Gaussian Naive bayes, k-NN, LR, RF, SVM	NA	accuracy = 0.97
				phosmet	2, 5, 8 s integration	spectrometer (QE-Pro)				
^41^	-	silver sol	drinking water	deltamethrin	3 s integration	DXR laser confocal microscopy Raman spectrometer (Thermo Fisher)	210	GRU	yes	accuracy = 0.98
								CNN		
								attention		
								ReLu		
^42^	0.001 ppb	COFs-Au@AgNPs	extra virgin oil	clothianidin	25 mW laser power	confocal Raman	>1215	improved informer	yes	accuracy = 1
				imidacloprid	2, 5, 8 s integration	spectrometer (WITec Alpha300)				
				acetamiprid						
^43^	0.1–1 ppm	CNA	river water	thiram	10 mW laser power	Raman spectrometer (Finder One, Beijing Zolix Instruments)	>200	SVM k-NN	NA	accuracy = 0.95
				carbendazim	10 s integration			decision tree		
				thiabendazole						
				carbaryl						
^44^	0.01 ppm (for thiabendazole)	Ag@BOCPs	orange juice	bromadiolone	1 s integration	confocal Raman	over billion	UMAP	NA	accuracy = 0.94
			blood	thiabendazole	20 scan	microscope (WITec alpha300R)		SVM		
			plasma serum	thiram	20 mW laser power			clustering analysis		
			pharmaceuticals	cypermethrin						
				acetamiprid						
				different tissue and blood proteins						
				pharmaceuticals						
^45^	1.2 ppm	AuAg alloyed NPs on paper filters	standard solution	thiram	1 s integration 5–20 mW laser power	Raman probe (AvaRaman-PRB-785)	240	DNN	yes	accuracy = 0.944
 ^46^	below MRL	stainless steel plate	standard solution (in water)	captan	10 s integration	confocal Raman	>2400	1D-CNN	NA	accuracy = 0.99
				tebuconazole	100 mW laser power	microscope (Senterra II				
				thiabendazole		spectrometer, Bruker Optics)				
^47^	19 ppm	Ag@Bs	pork meat	thiram veterinary drug residues	30 acquisitions	confocal Raman	-	MCR-ALS	yes	accuracy = 0.96
					1 s integration	microscope (WITec alpha300R)				
					30 mW laser power					
^48^	0.001 ppb	Ag@BO NPs	strawberry	organophosphorus-based	x power	Raman and SERS imaging microscopy integrated	>million	image processing	NA	-
			pears	dimethoate	5 s integration	system				
			apples	pyrethroid-based	1 scan					
			mango	cypermethrin						
			melon							
			tomato							
^49^	10 ppt	Ag@BN NPs	pericarp	thiabendazole	5 mW power	Raman and SERS imaging microscopy integratedsystem	>5000	CNN	yes	accuracy = 1
				acetamiprid	5 s integration					
				dimethoate	1 scan					
				glyphosate						
				chlorpyrifos						
				cypermethrin						
				azocyclotin						
This work	0.01 ppb for etoxazole and thiamethoxam	MIM-HCA array	cucumber	metalaxyl	100 mW laser power	custom-based Raman setup with 785 nm laser source	550	deep feed-forward neural networks	yes	accuracy = 0.927
				boscalid	2 s integration					
				famoxadone	25 scan					
				etoxazole						
				thiamethoxam						
				cypermethrin						

Table 2 summarizes representative studies on pesticide detection reported in the literature. Most of these studies report high accuracy (94–100%); the main reasons for this being (i) targeting a single or small number of analytes, (ii) performing measurements in standard solutions or relatively “clean” matrices, (iii) working with very large and balanced data sets, and (iv) using colloidal substrates with irregular hotspot distribution but aggressive in signal amplification. In contrast, the present study addresses a substantially more complex scenario: classification and quantification tasks assessed together on a real food matrix for six different pesticides on a MIM-HCA metasurface, with a limited number of data points (∼550 spectra). This context makes the reported 92.7% accuracy more meaningful, as many of the “perfect” accuracy claims in the comparison table were achieved under more favorable conditions in terms of problem complexity and data/substrate conditions.

## 4 Conclusion and Future Outlook

This study employed both qualitative and quantitative analysis of six different pesticides in the cucumber matrix on the same platform, integrating MIM-HCA metasurface analysis, a QuEChERS-based sample preparation method, an automated preprocessing pipeline (RamanPlot), and deep neural network models. The optical design was optimized to match the homogeneous “hotspot” distribution of the hexagonal structure and its structural resonance at 785 nm. This resulted in a stable SERS signal with a 2 s integration time and short measurement times of 20 scans.

The calibration curves generated for LoD accurately defined the dynamic range, realistically capturing the rapid increase in signal at low concentrations and saturation at high concentrations. The detection limits obtained were at or below the legal limits, demonstrating the measurement capability of the platform. In the ML component, which enables automated analyses, rigorous full-spectrum despiking, ARPLS baseline correction, Savitzky–Golay smoothing, and normalization with an internal standard were applied, thereby enhancing the classification ability of the model for both noisy matrix conditions and batch-to-batch variation. Classification accuracy was achieved at 92.7%, and for the more realistic 12-class data set scenario, which included blank cucumber matrices, the accuracy rate was 81%. On the data set targeting quantification for three characteristic concentration classes (10^–4^, 10^–7^, and 10^–9^ M), model accuracy reached 62.9%; specifically, the recall value at the lowest concentration level of 10^–9^ M was measured at 0.909, demonstrating strong model precision at this level. Most of the “near-perfect” accuracies frequently reported in the literature have been achieved using a small number of analytes, standard solutions, or clean matrices, as well as large and balanced data sets, and colloidal-based substrates with strong but more aggressive hotspots. In contrast, this study, which utilized a modest data set of ∼550 spectra, addressed the classification and quantification of six different pesticides within a real food matrix in the same scene. Thanks to the metasurface architecture, it achieved a production-friendly, reproducible, and interpretable SERS response down to subppb levels. This context highlights the significance of the reported 92.7% accuracy and the lower level of performance.

Regarding the drawbacks of this study, the small size of the data set affects its performance. The method should be strengthened with a larger number of examples and concentration ranges. To achieve this, multivariate regression-based quantification methods are also an aspect of the study that needs to be addressed and completed in future plans. Furthermore, combining the models into a multitasking model to enable the platform to evaluate more ML models simultaneously through a single model will increase the platform’s impact. However, this requires increasing the amount of data and reducing batch-to-batch variation. This is related to eliminating background noise at the production point. Furthermore, since real-world applications involve complex structures with multiple pesticide mixtures and chemicals, the lack of realistic multipesticide applications and other food matrix applications observed in this study is another shortcoming. These shortcomings are part of the future plans designed to improve this study. Despite all these shortcomings, the results demonstrate the applicability of the platform for agricultural surveillance in the field, with its reproducible metasurface architecture, monitoring matrix effects using QuEChERS, automated preprocessing, and short measurement times providing a practical basis for rapid screening (above/below MRL decisions) in the field or in storage.

For the further study plan, next steps should include validating equivalent performance with 785 nm portable/fiber-probe Raman systems at low power and short integration times; packaging MIM-HCA in disposable cartridge/strip format; and transitioning to scalable processes for mass production. Domain adaptation and calibration transfer (for instance Multiplicative Scatter Correction (MSC) and Standard Normal Variate (SNV) + batch-effect corrections) can bridge instrument/batch/laboratory differences, along with deep learning approaches that enable data sharing across different centers, thereby enhancing classification capability. In the context of the present MIM-HCA SERS platform, recent studies on group IVB transition metal nitrides (e.g., TiN, ZrN, and HfN) indicate that replacing noble metals with refractory plasmonic materials could improve long-term thermal and chemical stability without fundamentally altering the metasurface architecture. Such material systems may therefore represent a promising route for extending the operational lifetime and field robustness of MIM-based SERS platforms, particularly for agricultural and on-site applications.^50^

In conclusion, the AI-enabled MIM-SERS platform offers near-production, reproducible metasurface analysis, an automated and reproducible preprocessing pipeline, with subppb SERS performance and multiclass/layered ML outputs. It has proven to be a strong candidate for field applications in food safety inspection. Portable hardware integration, real-time software interfaces, product range extension, and model portability with federative learning enable the platform to scale to regulatory applications and widespread use cases.

## Supplementary Material



## Notes

SERS measurements of reference materials; data evaluation workflows for machine learning models; performance and quantification reports; description of RamanPlot GUI software; SERS spectra of pesticide combinations; structural characterization of hexagonal Au nanostructures; multilevel variance analysis; long-term signal stability assessment; comparison with EFSA maximum residue limits; spectroscopic ellipsometry analysis of PECVD-deposited SiNx layer (PDF)
