# Supplementary material for: Hexagonal Au Nanostructure SERS Metasurface for AI-Driven Detection of Pesticide Residues in Real Food Samples
Source: ACS Appl Nano Mater. 2026 Apr 10;9(16):7220–37. doi: 10.1021/acsanm.6c00433 (PMC13122584; doi:10.1021/acsanm.6c00433)
Supplement: Supplementary file 1 [file an6c00433_si_001.pdf]

Supporting Information

Hexagonal Au Nanostructure SERS Metasurface for AI-Driven Detection of Pesticide Residues in Real Food Samples

Sümeyra Vural Kaymaz[1], Mustafa Özen[2], Süleyman Çelik[3], Selim Tanrıseven[3], Elmas Eva Öktem Olgun[4], Oltan Canlı[4], Barış Güzel[4], Yunus Sarıkaya[5], Hasan Kurt[6, 7, 8, *], Meral Yüce[3, 8*]

[1] Department of Molecular Biology, Genetics, and Bioengineering, Faculty of Engineering and Natural Sciences, Sabanci University, 34956, Istanbul, Türkiye

[2] Department of Computer Science, Faculty of Engineering and Natural Sciences, Sabanci University, 34956, Istanbul, Türkiye

[3] SUNUM Nanotechnology Research and Application Centre, Sabanci University, Istanbul, 34956, Türkiye

[4] Climate Studies and Water Management Research Group, Climate and Life Sciences Vice Presidency, TUBITAK Marmara Research Center, 41470 Gebze, Kocaeli, Türkiye

[5] Pusula AI, Oakland, CA, 94611, USA

[6] Department of Biomedical Engineering, School of Engineering and Natural Sciences, Istanbul Medipol University, Istanbul, 34810, Türkiye

[7] Research Institute for Health Sciences and Technologies (SABITA), Istanbul Medipol University, Istanbul, 34810, Türkiye

[8] Department of Bioengineering, Imperial College London, South Kensington Campus, London, SW7 2AZ, UK

***Corresponding authors**: h.kurt@imperial.ac.uk also hasankurt@medipol.edu.tr and meralyuce@sabanciuniv.edu

Table of Contents

Hexagonal Au Nanostructure SERS Metasurface for AI-Driven Detection of Pesticide Residues in Real Food Samples 1

1. **SERS Measurements of Reference Materials** 3
2. **Data Evaluation for ML Models** 4
3. **Performance and Quantification Reports for ML Model 2** 5
4. **RamanPlot GUI software** 8
5. **SERS spectra of pesticide combinations** 8
6. **Characterization of the hexagonal Au nanostructure** 11
7. **Multilevel Variance Analysis of Hexagonal Au Nanostructures** 12
8. **Long-term Signal Stability Check** 12
9. **Comparison with EFSA MRLs** 13
10. **Spectroscopic ellipsometry analysis of the PECVD-deposited SiNx layer** 14

1. SERS Measurements of Reference Materials

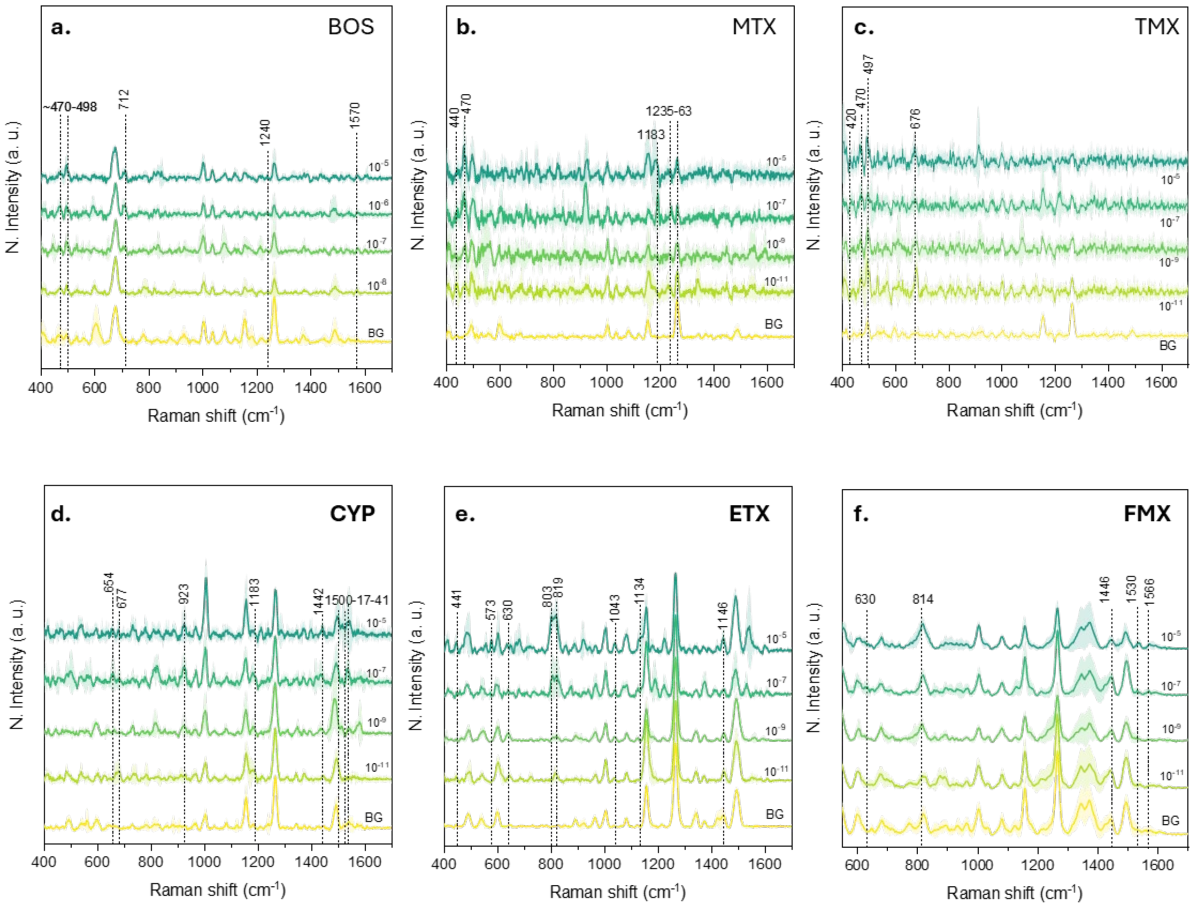

Figure S1. SERS spectra of 6 pesticides as reference material.

2. Data Evaluation for ML Models

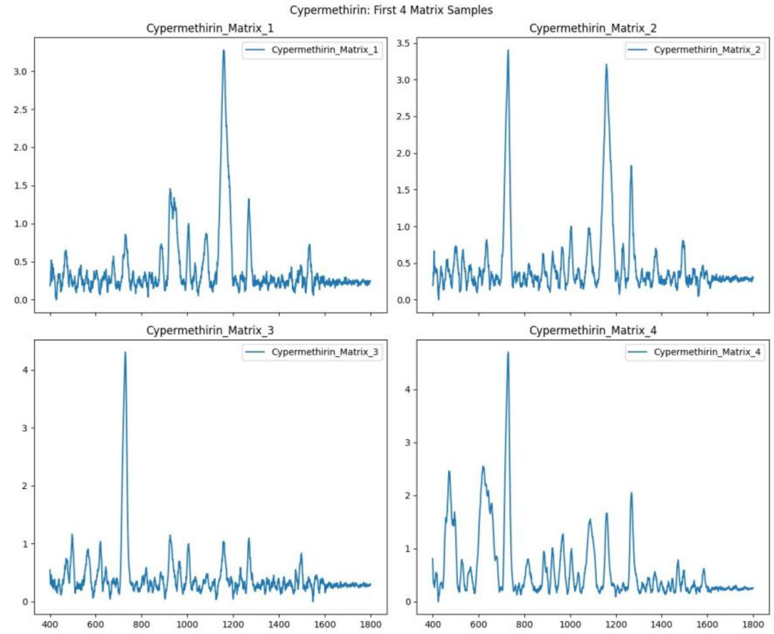

Figure S2. The first four SERS spectra of the pesticide cypermethrin obtained from different matrix samples. *All spectra belong to the same class (cypermethrin), with minor variations in peak intensity and position due to experimental conditions and matrix effects.*

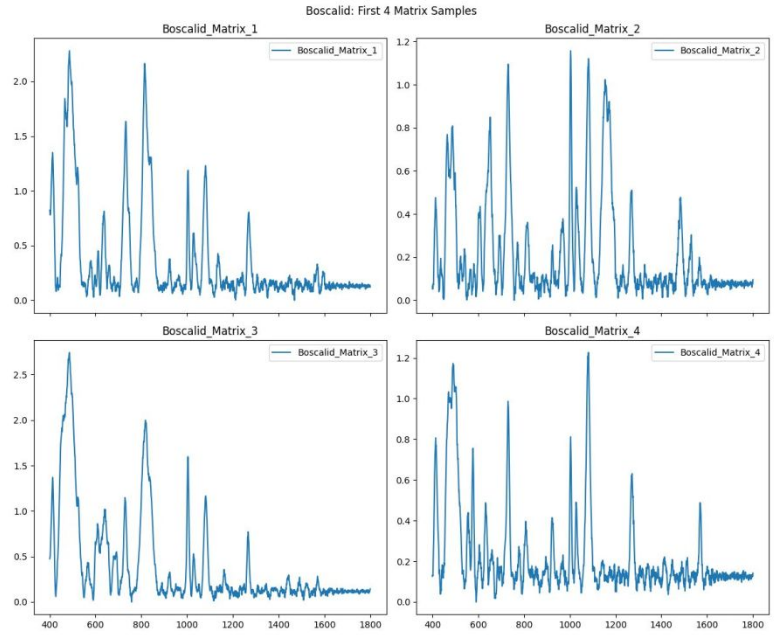

Figure S3. The first four SERS spectrum of Boscalid pesticide was obtained from different matrix samples. *Although they belong to the same class, variations in signal intensity and patterns are observed.*

3. Performance and Quantification Reports for ML Model 2

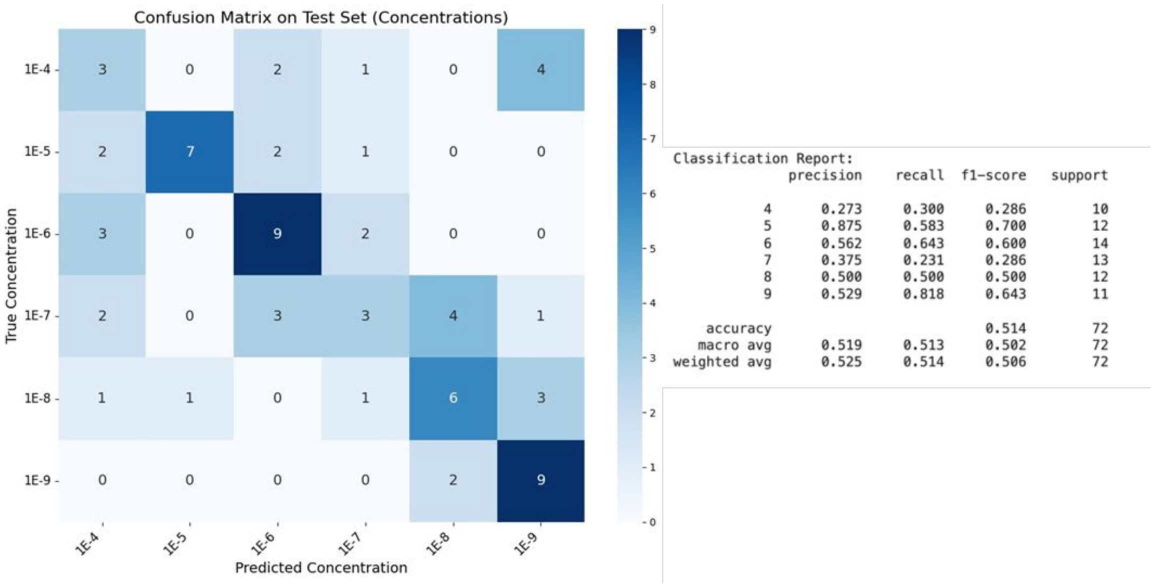

Figure S4. Performance report of the model used in the classification of six different concentration levels. *The overall accuracy of the model was 51%, with better results, particularly in the mid-range concentration range (e.g., around* 10^{-6} *M). The balanced precision and recall values indicate that the model generalizes correctly for certain classes but struggles at low signal intensities. Confusion matrix generated by concentrations in the test set. While a high correct classification rate was observed at the* 10^{-6} *M concentration, inter-class confusion was evident at high (*10^{-4} *M) and low (*10^{-8}*–*10^{-9} *M) concentrations. This is due to the low signal-to-noise ratio of low-intensity spectra and spectral similarities.*

Table S1. Quantification report for the results shown in confusion matrix in Figure 4.

Pesticide	*Replicate*	*true_concentration_str*	*predicted_concentration_str*
TMX	*1*	*1E-5*	*1E-6*
BOS	*15*	*1E-7*	*1E-8*
FMX	*6*	*1E-8*	*1E-8*
MTX	*3*	*1E-5*	*1E-5*
MTX	*5*	*1E-7*	*1E-7*
CYP	*9*	*1E-5*	*1E-5*
MTX	*10*	*1E-7*	*1E-8*
BOS	*6*	*1E-7*	*1E-4*
MTX	*12*	*1E-6*	*1E-6*
TMX	*9*	*1E-9*	*1E-8*
TMX	*3*	*1E-5*	*1E-5*

ETX	*3*	*1E-9*	*1E-9*
BOS	*1*	*1E-6*	*1E-4*
TMX	*12*	*1E-9*	*1E-8*
FMX	*5*	*1E-5*	*1E-6*
ETX	*8*	*1E-8*	*1E-9*
ETX	*1*	*1E-7*	*1E-6*
ETX	*8*	*1E-4*	*1E-9*
ETX	*8*	*1E-6*	*1E-6*
ETX	*10*	*1E-7*	*1E-9*
BOS	*11*	*1E-7*	*1E-8*
CYP	*7*	*1E-6*	*1E-6*
TMX	*12*	*1E-5*	*1E-7*
FMX	*8*	*1E-7*	*1E-7*
ETX	*10*	*1E-4*	*1E-9*
CYP	*1*	*1E-9*	*1E-9*
MTX	*8*	*1E-7*	*1E-8*
TMX	*13*	*1E-6*	*1E-6*
CYP	*8*	*1E-4*	*1E-4*
TMX	*5*	*1E-7*	*1E-7*
MTX	*1*	*1E-6*	*1E-6*
ETX	*11*	*1E-6*	*1E-6*
TMX	*6*	*1E-8*	*1E-9*
CYP	*4*	*1E-9*	*1E-9*
MTX	*5*	*1E-9*	*1E-9*
MTX	*7*	*1E-6*	*1E-4*
FMX	*9*	*1E-5*	*1E-5*
ETX	*6*	*1E-9*	*1E-9*
TMX	*1*	*1E-6*	*1E-7*
CYP	*6*	*1E-8*	*1E-8*
BOS	*10*	*1E-7*	*1E-6*
BOS	*7*	*1E-4*	*1E-4*

ETX	*3*	*1E-6*	*1E-6*
TMX	*11*	*1E-5*	*1E-5*
FMX	*7*	*1E-8*	*1E-8*
BOS	*15*	*1E-5*	*1E-5*
MTX	*7*	*1E-8*	*1E-8*
TMX	*2*	*1E-6*	*1E-7*
TMX	*1*	*1E-4*	*1E-6*
BOS	*3*	*1E-6*	*1E-4*
BOS	*5*	*1E-7*	*1E-4*
MTX	*9*	*1E-5*	*1E-4*
MTX	*11*	*1E-5*	*1E-5*
FMX	*9*	*1E-4*	*1E-7*
MTX	*4*	*1E-8*	*1E-4*
CYP	*9*	*1E-8*	*1E-8*
ETX	*5*	*1E-4*	*1E-9*
CYP	*9*	*1E-6*	*1E-6*
MTX	*2*	*1E-6*	*1E-6*
ETX	*6*	*1E-7*	*1E-6*
CYP	*2*	*1E-8*	*1E-5*
ETX	*10*	*1E-9*	*1E-9*
TMX	*1*	*1E-9*	*1E-9*
FMX	*4*	*1E-5*	*1E-4*
CYP	*1*	*1E-4*	*1E-4*
ETX	*7*	*1E-8*	*1E-7*
TMX	*17*	*1E-9*	*1E-9*
CYP	*7*	*1E-9*	*1E-9*
MTX	*13*	*1E-8*	*1E-8*
MTX	*2*	*1E-8*	*1E-9*
MTX	*2*	*1E-4*	*1E-6*
ETX	*1*	*1E-4*	*1E-9*

4. RamanPlot GUI software

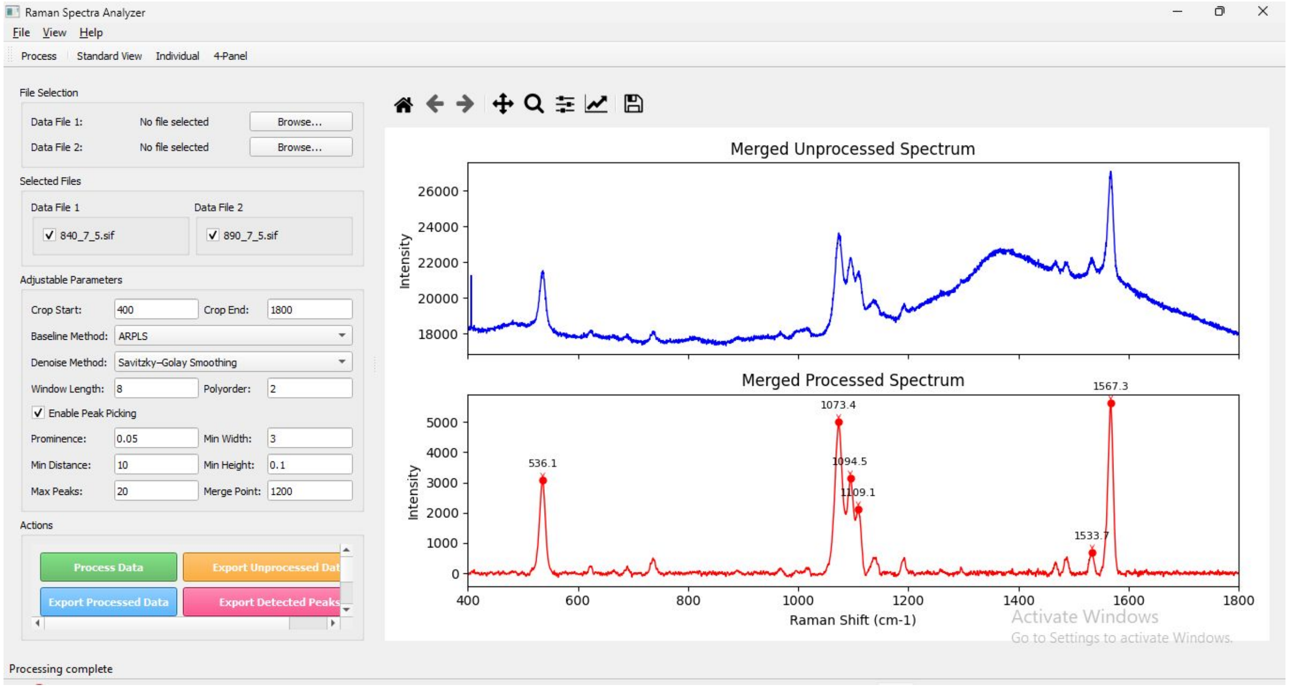

Figure S5. Snapshot taken from RamanPlot GUI software.

5. SERS spectra of pesticide combinations

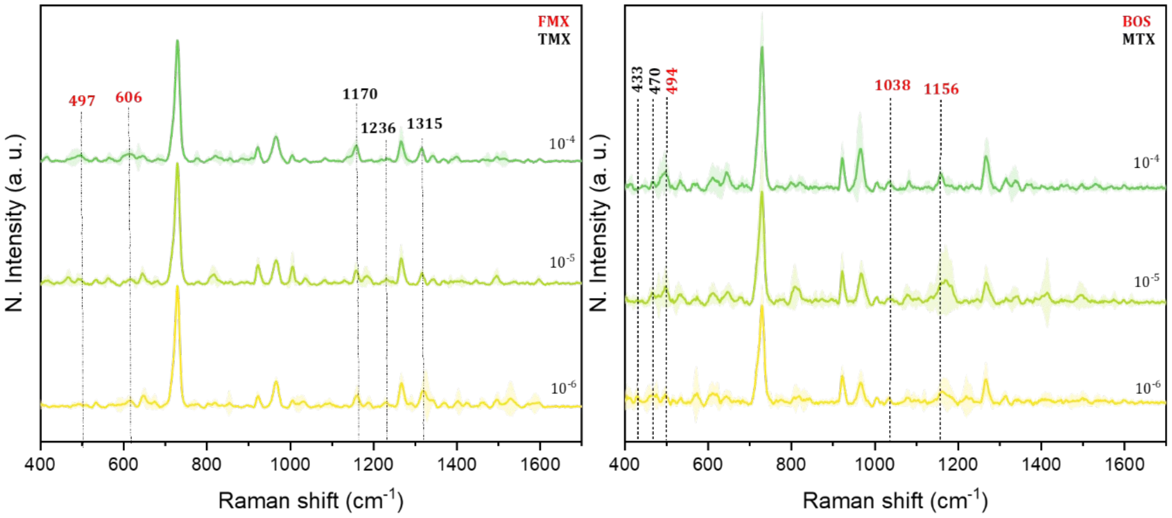

Figure S6. SERS spectra of pesticide combinations in cucumber matrix.

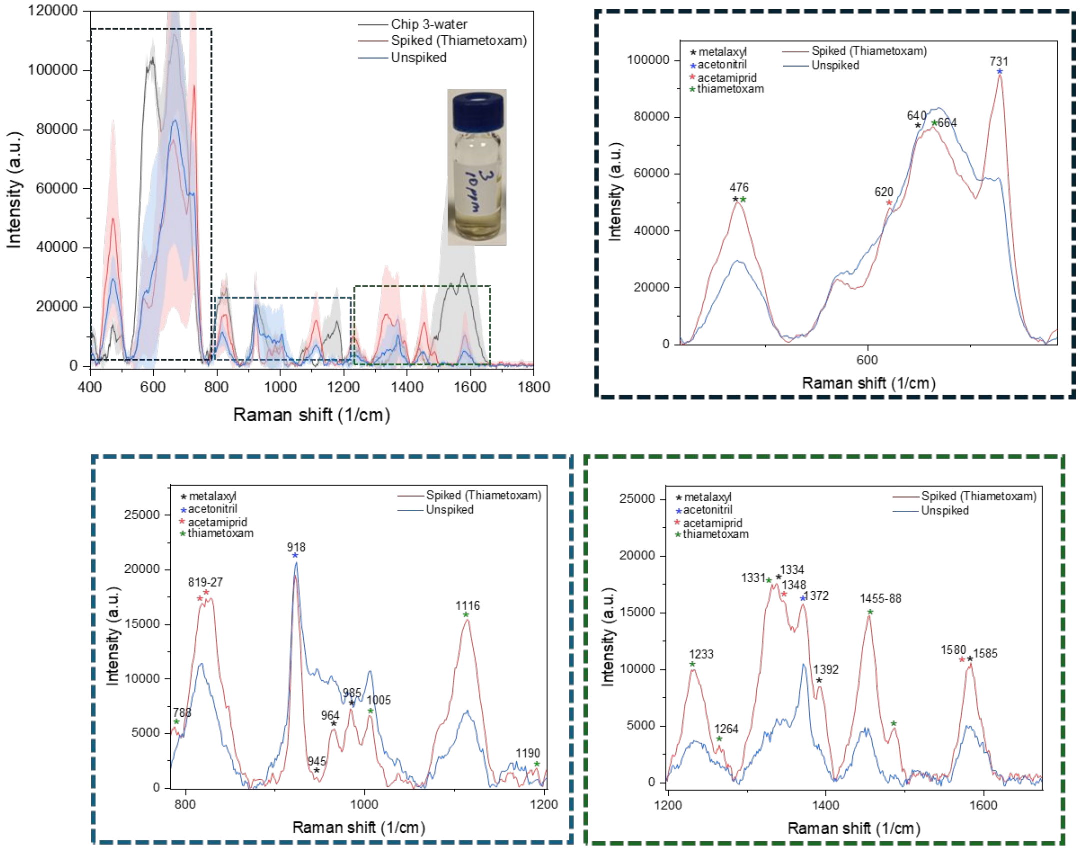

Figure S7. SERS spectra of pesticide combinations.

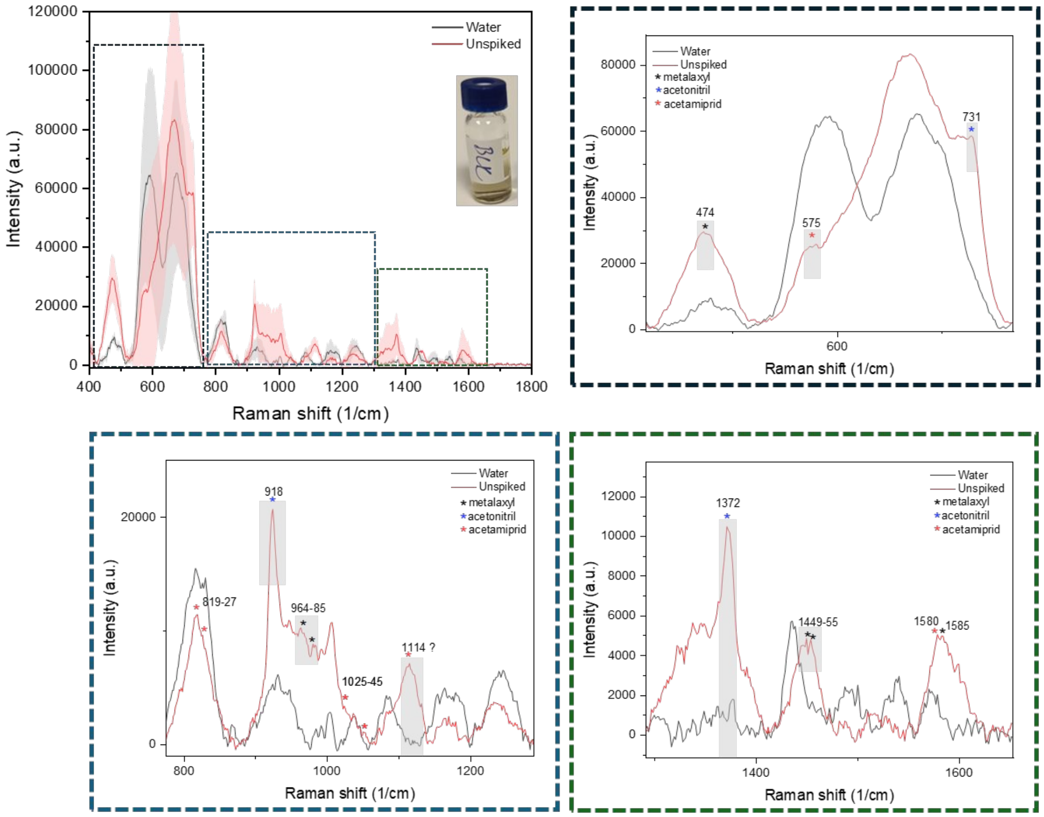

Figure S8. SERS spectra of unspiked (naturally contaminated) sample.

6. Characterization of the hexagonal Au nanostructure

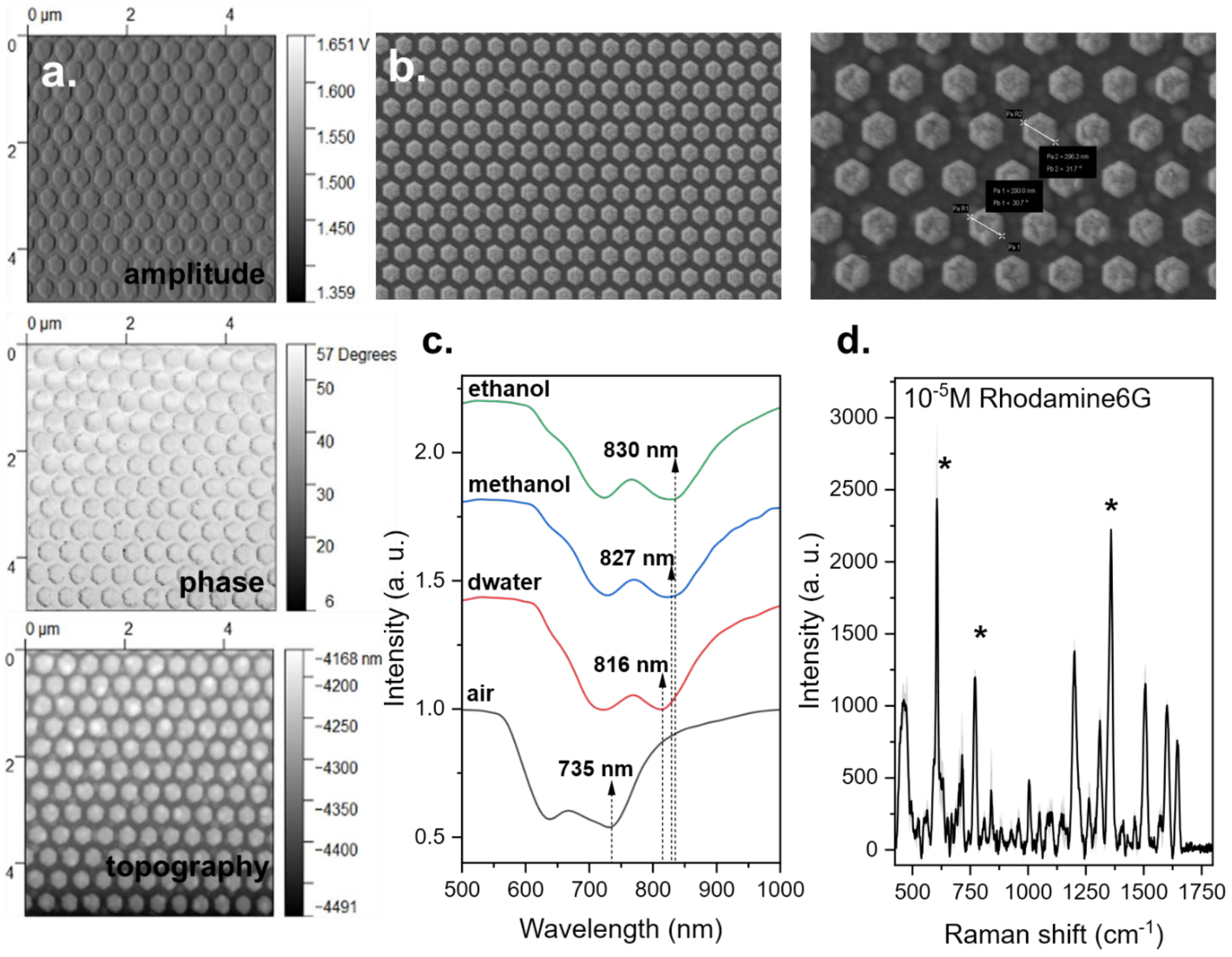

Figure S9. Morphological, topographical, and optical characterization of the hexagonal Au nanostructure metasurface. *(a) AFM amplitude image showing a highly ordered hexagonal array over a 5 × 5 μm² area. AFM phase image highlighting material contrast and uniform surface response. AFM topography image revealing consistent height distribution and well-defined nanostructure profiles. (b) SEM image confirming large-area periodicity and uniform hexagonal packing of the nanostructures and high-magnification SEM image used to extract geometric parameters (pitch and feature size), demonstrating low structural variation across the array. (f) Representative optical response (reflection spectra) of the metasurface, indicating plasmonic resonance features in different refractive index medium relevant to the excitation (around 785 nm) and Stokes scattering regions. (g) Representative SERS spectrum acquired from the metasurface, demonstrating signal enhancement of the substrate with Rhodamine6g Raman reporter with 785 nm laser source and 20 mW laser power with 30 accumulations/ 1 s integration.*

7. Multilevel Variance Analysis of Hexagonal Au Nanostructures

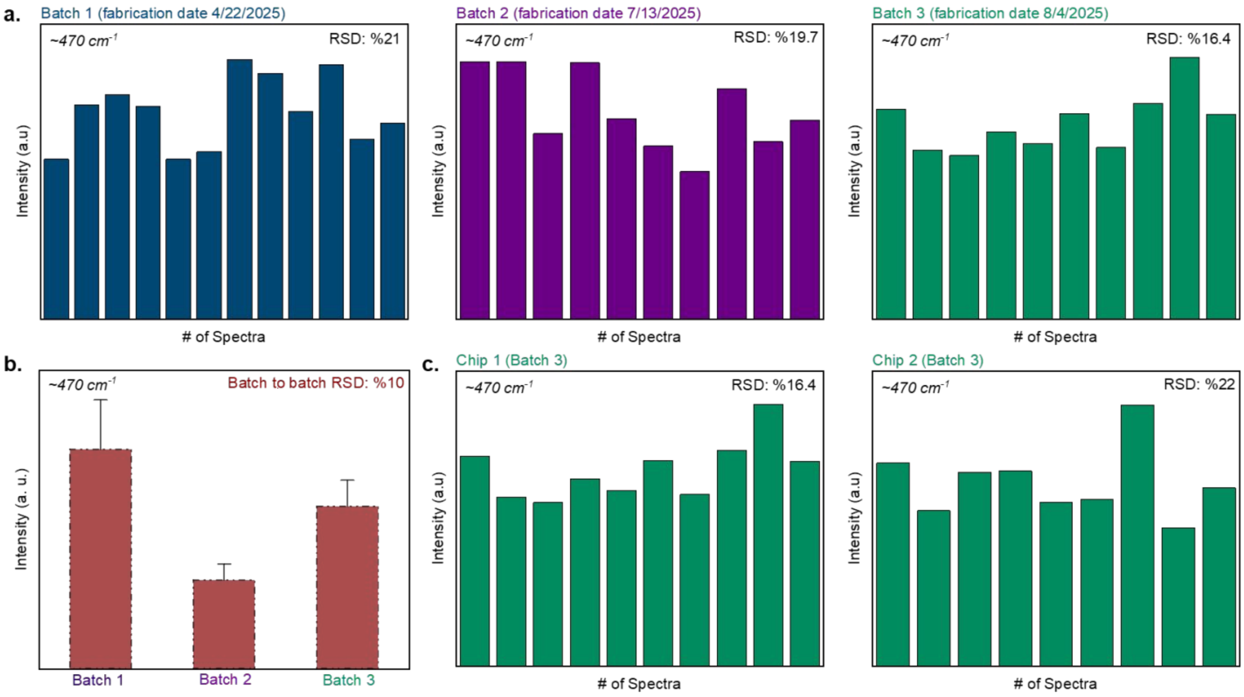

Figure S10. Multilevel variance analysis of the SERS response using Metalaxyl as a representative analyte. *(a) Spot-to-spot SERS intensity distributions obtained from three independently fabricated batches (Batch 1: 4/22/2025, Batch 2: 7/13/2025, Batch 3: 8/4/2025), where each bar represents an individual measurement spot on a single chip (n = 10 spots per chip).(b) Batch-to-batch variability quantified by the relative standard deviation (RSD) of the ensemble-averaged SERS intensities across the three batches (n = 3 batches). (c) Chip-to-chip variability evaluated within Batch 3 by comparing two independently fabricated chips, each measured at 10 distinct spatial locations (n = 2 chips). All measurements were performed using Metalaxyl at a concentration of* 10^{-5} *M under 785 nm laser excitation with a laser power of 100 mW, an integration time of 1 s, and 30 accumulations. Ensemble averages were calculated hierarchically by first averaging intensities across spots within each chip, followed by averaging across chips where applicable.*

8. Long-term Signal Stability Check

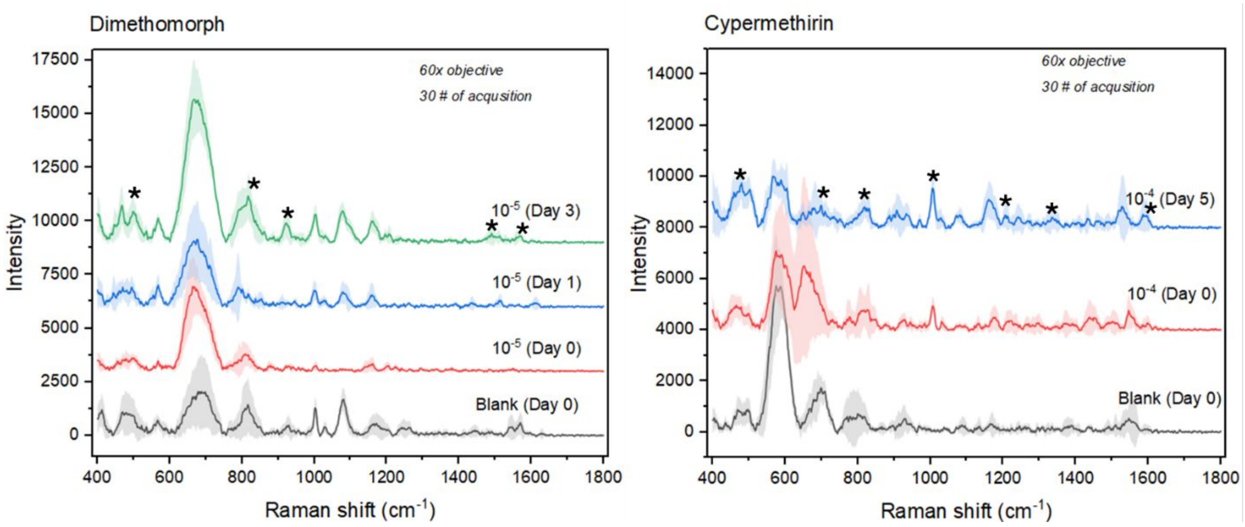

Figure S11. Long-term signal stability of the MIM-based SERS substrate evaluated using representative pesticide analytes. *Time-dependent SERS spectra of dimethomorph (left, 10^{-5} M) and cypermethrin (right, 10^{-4} M) recorded on the same substrate at different time points (Day 0, Day 1, Day 3 for dimethomorph; Day 0 and Day 5 for cypermethrin). Measurements were performed using a 60× objective with 30 spectral acquisitions under identical experimental conditions by the same user. Characteristic Raman bands (marked by asterisks) remain clearly identifiable over time without noticeable peak shifts or signal attenuation, demonstrating the stability of the plasmonic substrate. Shaded regions represent the standard deviation obtained from repeated measurements.*

9. Comparison with EFSA MRLs

Table S2. Stepwise conversion of sensor LoDs from mol/L in extract to equivalent concentrations in cucumber (mg/kg, ppm), including comparison with EFSA MRLs.

Parameter	MTX	BOS	FMX	CYP	TMX	ETX
Sensor LOD in extract (mol/L)	4.80E-07	1.60E-06	3.70E-09	6.34E-07	6.80E-08	9.80E-08
Molecular weight (g/mol)	279.33	343.21	374.4	416.3	291.71	359.4
Concentration in extract (mg/L)	1.34E-01	5.49E-01	1.39E-03	2.64E-01	1.98E-02	3.52E-02
Extract volume (L)	0.01	0.01	0.01	0.01	0.01	0.01
Total mass in extract (mg)	1.34E-03	5.49E-03	1.39E-05	2.64E-03	1.98E-04	3.52E-04
Cucumber sample mass (kg)	0.01	0.01	0.01	0.01	0.01	0.01
Equivalent concentration in cucumber (mg/kg)	1.34E-01	5.49E-01	1.39E-03	2.64E-01	1.98E-02	3.52E-02
Equivalent concentration in cucumber (ppm)	0.1 ppm	0.5 ppm	0.001 ppm	0.2 ppm	0.01 ppm	0.03 ppm
EFSA MRL (cucumber) (mg/kg)	0.5	4	0.2	0.2	0.01	0.01

10. Spectroscopic ellipsometry analysis of the PECVD-deposited SiNx layer

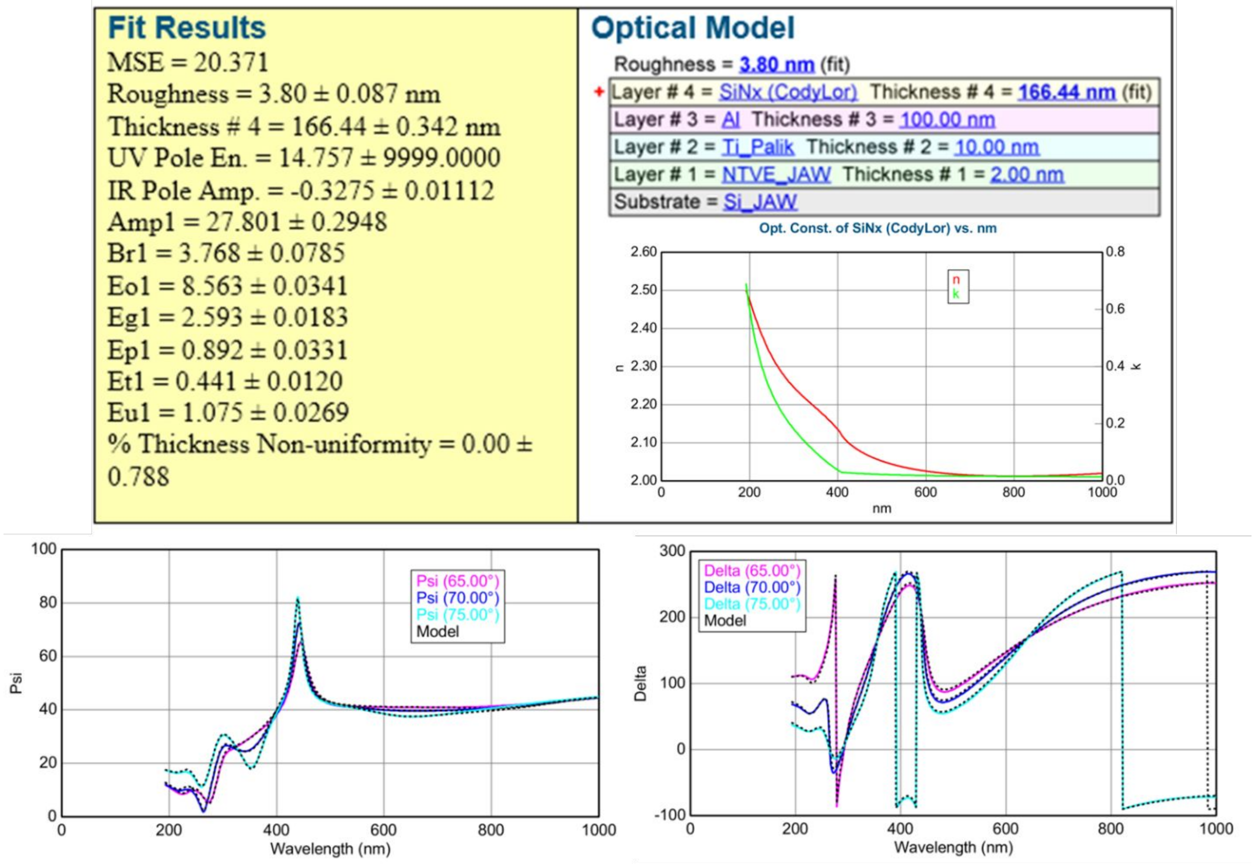

Figure S12. Spectroscopic ellipsometry analysis of the PECVD-deposited SiNx layer on the MIM substrate. *The optical model consists of a Si substrate, native oxide, Ti adhesion layer (10 nm), Al layer (100 nm), and a top SiNx layer. Simultaneous fitting of Ψ and Δ spectra acquired at three incidence angles (65°, 70°, and 75°) yields a SiNx thickness of 166.4 ± 0.3 nm with a surface roughness of 3.8 ± 0.1 nm. The excellent agreement between experimental data and model confirms the reliability of the fitted thickness and optical constants.*
